# Range‐Wide Genomic Analysis Reveals Regional and Meta‐Population Dynamics of Decline and Recovery in the Grey Seal

**DOI:** 10.1111/mec.17824

**Published:** 2025-06-19

**Authors:** Morgan L. McCarthy, Kristina M. Cammen, Sandra M. Granquist, Rune Dietz, Jonas Teilmann, Charlotte Bie Thøstesen, Simon Kjeldgaard, Mia Valtonen, Mervi Kunnasranta, Bjørn Munro Jenssen, Markus P. Ahola, Britt‐Marie Bäcklin, W. Don Bowen, Wendy B. Puryear, Jonathan A. Runstadler, Debbie J. F. Russell, Anders Galatius, Morten Tange Olsen

**Affiliations:** ^1^ Section for Molecular Ecology and Evolution, Globe Institute University of Copenhagen Copenhagen K Denmark; ^2^ School of Marine Sciences University of Maine Orono Maine USA; ^3^ The Icelandic Seal Center Hvammstangi Iceland; ^4^ Marine and Freshwater Research Institute Hafnarfjörður Iceland; ^5^ Section for Marine Mammal Research, Department of Ecoscience Aarhus University Roskilde Denmark; ^6^ Museum VEST Ribe Denmark; ^7^ Department of Environmental and Biological Sciences University of Eastern Finland Joensuu Finland; ^8^ Department of Biology Norwegian University of Science and Technology Trondheim Norway; ^9^ Department of Population Analysis and Monitoring Swedish Museum of Natural History Stockholm Sweden; ^10^ Marine Environment Research Group Turku University of Applied Sciences Turku Finland; ^11^ Department of Environmental Monitoring and Research Swedish Museum of Natural History Stockholm Sweden; ^12^ Department of Biology, Life Science Centre Dalhousie University Halifax Nova Scotia Canada; ^13^ Department of Infectious Disease and Global Health, Cummings School of Veterinary Medicine Tufts University North Grafton Massachusetts USA; ^14^ Sea Mammal Research Unit, School of Biology University of St Andrews St Andrews Fife UK

**Keywords:** conservation genomics, *Halichoerus grypus*, hunting, management units, marine mammals, recovery, top‐predator

## Abstract

Wildlife populations globally have experienced widespread historical declines due to anthropogenic and environmental impacts, yet for some species, contemporary management and conservation programmes have enabled recent recovery. The impacts of decline and recovery on genomic diversity and, vice versa, the genetic factors that contribute to conservation success or failure are rich areas for inquiry, with implications for shaping how we manage species into the future. To comprehensively characterise these processes in natural systems requires range‐wide sampling and international collaboration, particularly for species with wide dispersal capabilities, broad geographic distributions, and complex regional metapopulation dynamics. Here, we present the first range‐ and genome‐wide population genomic analysis of grey seals based on 3812 nuclear SNPs genotyped in 188 samples from 17 localities. Our analyses support the existence of three main grey seal populations centred in the NW Atlantic, NE Atlantic and Baltic Sea, and point to the existence of previously unrecognised substructure within the NE Atlantic. We detected remarkably low levels of genetic diversity in the NW Atlantic population, and demographic analyses revealed a turbulent history of NE Atlantic and Baltic Sea grey seals, with bottlenecks in the Middle Ages and the 20th century due to hunting and habitat alterations. We found some localities deviated from isolation by distance patterns, likely reflecting wide‐scale metapopulation dynamics associated with recolonisation and recovery in regions where they were historically extirpated. We identify at least six grey seal genetic populations and reveal marked genetic effects of past declines and recent recovery across the species' range.

## 1 Introduction

Earth ecosystems are increasingly impacted by human activities and climate change, leading to widespread declines in intra‐ and interspecific organismal diversity (Luypaert et al. 2020; Exposito‐Alonso et al. 2022; Richardson et al. 2023). A valuable—yet often poorly communicated—lesson from recent conservation and management efforts is that it is possible to turn the tide if species are adequately protected and given space and time to recover. Such conservation successes have been recorded across marine and terrestrial ecosystems, including among mammals (Punt and Wade 2010; Pearson et al. 2025; Godoy et al. 2025), birds (Li et al. 2014), fish (Rosenberg et al. 2006), reptiles (Chaloupka et al. 2008), and plants (Hughes et al. 2012). However, many more species have failed to recover or just started their decline, raising questions about the underlying ecological and/or genetic factors that contribute to conservation success or failure.

Population genomic diversity is a prerequisite for adaptation to human and environmental impacts, and is in itself a rich source of data, readily accessed today through modern sequencing technology. From genomic data, we can infer the recent historical past of diverse species and characterise contemporary population dynamics, including levels of diversity, isolation and connectivity (e.g., Waples and Gaggiotti 2006; Palsbøll et al. 2007). Critical to effective conservation and management, these types of data can inform the appropriate spatiotemporal scale at which to assess current human and environmental impacts, as well as recovery from past impacts and potential responses to those predicted to occur in the future. However, comprehensively characterising the population genomic diversity of a species can be challenging; differences in geographic sampling extent and/or molecular methods may yield conflicting results, or tell only part of the story (e.g., Cunha et al. 2014; Cabrera et al. 2019; Pedraza‐Marrón et al. 2019; Stankiewicz et al. 2021). Conducting range‐wide population genomic analyses, though logistically challenging, should therefore be the ideal aim of any conservation genomic study, particularly for species with wide dispersal capabilities, broad geographic distributions, and complex regional metapopulation dynamics (Liu et al. 2022; McCarthy et al. 2025). Range‐wide analyses can detect patterns not revealed in regional or local studies, reveal new populations and ecotypes and uncover cryptic barriers to dispersal (de Jong et al. 2023; Rexer‐Huber et al. 2019; Rosing‐Asvid et al. 2023). For species whose ranges span national boundaries, range‐wide analyses can also serve as a cornerstone for developing collaborative international management approaches (Attard et al. 2024; Gose et al. 2024; Miller et al. 2024).

The grey seal ( *Halichoerus grypus* ) is a wide‐ranging, broadly distributed pinniped species, occurring in temperate and subarctic regions of the North Atlantic and adjoining seas. The species often embarks on long‐distance foraging trips and uses several distinct land‐based resting and moulting sites, yet typically exhibits some level of natal site‐fidelity during the breeding season, lending itself to complex metapopulation dynamics (McConnell et al. 1999; Pomeroy et al. 2000; Karlsson et al. 2005; Russell et al. 2013; Brasseur et al. 2015; Vincent et al. 2017; Carter et al. 2020). It is also a species with a tumultuous history of human exploitation and protection that has resulted in drastic changes in population size. Grey seals suffered severe population declines and multiple local extirpations due to high historical hunting and culling pressure in the 19th and 20th centuries, but in areas where protections are now in place, the species has demonstrated a remarkable ability to recover and recolonise former haul‐out sites (Härkönen et al. 2007; Brasseur et al. 2015; Fietz et al. 2016; Olsen et al. 2018; Wood et al. 2020; Galatius et al. 2020, 2024; den Heyer et al. 2021), and in some cases establish new haul‐out sites for resting, moulting and/or breeding (Russell et al. 2019). While a story of conservation success, it also has implications for emerging interspecific conflicts, including with humans, fisheries, and other protected species, such as sympatric harbour porpoises ( *Phocoena phocoena* ) and harbour seals ( *Phoca vitulina* ) (van Neer et al. 2015; Olsen et al. 2018; Cammen et al. 2019; Thompson et al. 2019), as well as implications for emerging pathogens, given the potential role of grey seals as disease reservoirs (Puryear et al. 2016). Furthermore, as grey seal populations grow and expand, currently recognised genetic population structure, levels of diversity and delineations of Management Units (MUs), Evolutionary Significant Units (ESUs), Distinct Population Segments (DPSs) and subspecies may shift (Fietz et al. 2016; Galatius et al. 2024). In the light of all this, an updated assessment of grey seal population genomic diversity—utilising genome‐wide SNPs and a near‐complete coverage of the species' range—is timely.

The grey seal has been subject to multiple prior genetic studies at global and local scales based on variation in the mitochondrial (mtDNA) control region and/or nuclear microsatellite and restriction site‐associated DNA sequencing (RADseq) loci. From these studies, it is generally accepted that across its range, the grey seal comprises at least three main genetic populations in the Northwest (NW) Atlantic, Northeast (NE) Atlantic and Baltic Sea, respectively (Boskovic et al. 1996; Klimova et al. 2014). However, the taxonomic status of these regional groups has been debated; previously proposed as three subspecies (Nehring 1886; Chapskii 1975), today only two subspecies are recognised by the IUCN and Society for Marine Mammalogy: *H. grypus atlantica* spanning the NW and NE Atlantic populations, and *H. grypus grypus* in the Baltic Sea (Berta and Churchill 2012; Olsen et al. 2016; Galatius et al. 2022). Contributing to this confusion, prior studies have reported that the most genetically dissimilar population pairs are the NW Atlantic and Baltic Sea grey seals for nuclear microsatellite loci, but the NW Atlantic and NE Atlantic grey seals for mtDNA (Boskovic et al. 1996; Klimova et al. 2014). At a regional scale, genetic studies have also yielded conflicting results with regards to the level of genetic differentiation among and within regions; while there is little to no genetic differentiation among NW Atlantic grey seals (Wood et al. 2011; Cammen, Schultz, et al. 2018), fine‐scale structure has been reported within the NE Atlantic and Baltic Sea by some, but not all studies (Allen et al. 1995; Gaggiotti et al. 2002; Graves et al. 2009; Fietz et al. 2013; Klimova et al. 2014; Fietz et al. 2016; Decker et al. 2017; Steinmetz et al. 2024).

Synthesising a single understanding of the grey seal's population structure and bridging population dynamics from the local to the range‐wide scale from these diverse studies have been challenging. As a result, our understanding of the species' recent history is incomplete, and our ability to assess diversity, demographic trends and metapopulation dynamics is limited. Here, we therefore conducted a range‐ and genome‐wide analysis of grey seals to (1) characterise contemporary population structure, including estimating the degree of isolation and connectivity within and between regions, and (2) infer recent demographic history and genomic diversity to contextualise past and present‐day population size trends. Despite the logistical and economic challenges often associated with embarking on international range‐wide population genomic analyses, our study should be inspirational for other efforts aimed at widely dispersed species characterised by a turbulent demographic history, local metapopulation dynamics, cryptic dispersal barriers and other sources of spatial heterogeneity in genetic diversity.

## 2 Materials and Methods

### 2.1 Sampling and DNA Extraction

**FIGURE 1:**
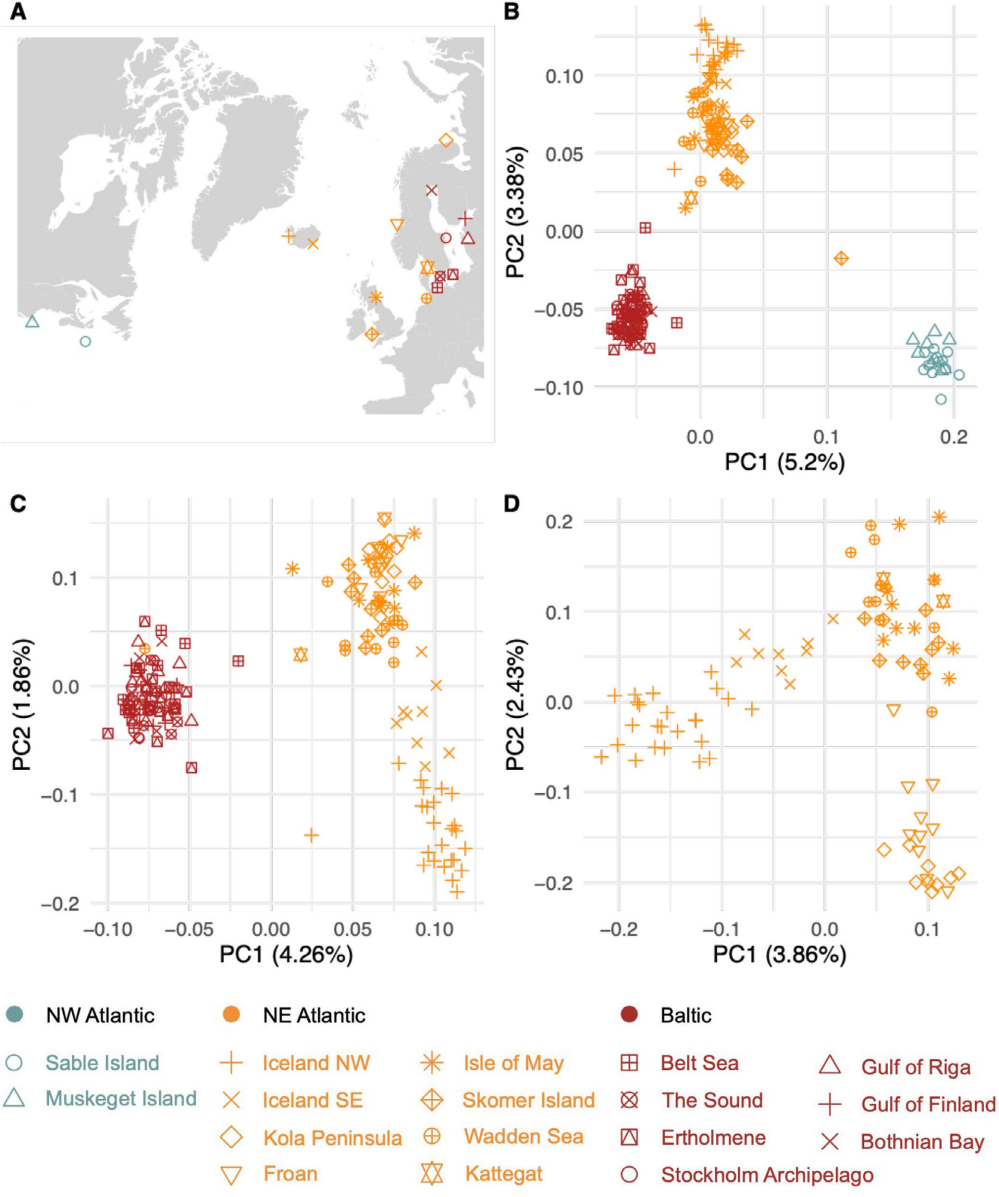
Range‐wide population genomic structure of the grey seal inferred by PCA. (A) Map of sampling localities; (B) Range‐wide population structure for PC1‐PC2, suggesting three main genetic clusters; (C) PC1‐PC2 for Northeast Atlantic and Baltic populations only; (D) PC1‐PC2 for Northeast Atlantic populations only. See Figures S7–S10 for additional PC axes. The fraction of total variation in the dataset explained for each PC is provided as a percentage. Localities within a broader region are colour coded with blue from the Northwest Atlantic, orange from the Northeast Atlantic, and red from the Baltic Sea.

Long‐term, dedicated sampling efforts allowed us to obtain a total of 222 grey seal samples from 17 distinct localities across the North Atlantic range of the species (Figure 1A; Figure S1; Table S1). For ease of analysis, interpretation and visualisation, multiple samples originating from the same specific site are grouped by locality (e.g., Ertholmene), whereas multiple single samples originating from geographically proximate sites are grouped by region (e.g., Kattegat). DNA extractions were performed in laboratories at the University of Copenhagen and the University of Maine using the Qiagen DNeasy Blood & Tissue kit or Thermo Fisher Scientific KingFisher Cell and Tissue DNA kit following the manufacturer's instructions. To facilitate tissue digestion, 20 μL of 1 M DTT (Sigma) was added during the digestion step to extractions performed using the Qiagen DNeasy Blood & Tissue kit. DNA quality and quantity were assessed on 1% agarose gels with a 1 kb ladder and on a Qubit Fluorometer, respectively.

### 2.2 ddRADseq Library Preparation and Sequencing

The samples were sequenced using double‐digest restriction site‐associated DNA sequencing (ddRADseq) following Peterson et al. (2012), with minor modifications described in Cammen, Schultz, et al. (2018). ddRADseq libraries were generated at the Duke University Marine Conservation Molecular Facility. Briefly, genomic DNA was double‐digested using SBfI‐HF and MspI restriction enzymes (New England Biolabs). Digested samples were uniquely barcoded and approximately evenly distributed in pools of 32 individuals per library across 10 libraries to minimise batch effects. Library sequencing was performed by the Duke Center for Genomic and Computational Biology on five lanes of an Illumina HiSeq 2500 utilising 50 bp single‐end read sequencing.

### 2.3 Mapping

Raw reads were demultiplexed with the process_radtags function from STACKS v.2.61 (Catchen et al. 2013). Following Liu et al. (2022), demultiplexed reads were trimmed for low‐quality bases and adaptor sequences with fastp v.0.22 (Chen et al. 2018), using a minimum value of 15 for qualified bases (‐q 15), a maximum of 50% unqualified bases allowed per read (‐u 50), and a sliding window size of 5 bases (‐W 5). Pruned reads were mapped to the repeat masked chromosome‐length grey seal genome assembly from DNAZoo (www.dnazoo.org) (Smit et al. 2015; Dudchenko et al. 2017) using the BWA‐MEM algorithm (Li and Durbin 2009) with default settings.

### 2.4 Data Quality Filtering

We took an iterative approach for filtering samples and low‐quality genomic regions. Using an initial set of genotype likelihoods called across all samples, we removed samples that were misidentified during sampling or storage. After confirming species IDs, we removed samples that were either duplicates or related individuals. Genomic region filtering included omitting sex chromosomes, loci that were flagged as out of Hardy–Weinberg Equilibrium (HWE), and loci on the tails of the depth distribution (10th and 90th percentile) across all samples remaining in the datasets.

### 2.5 Sample Quality Filtering

The average depth of coverage (×) and standard deviation for our ddRADseq dataset was calculated for each sample using SAMtools v.1.12 (Li et al. 2009). Samples with an average depth less than three were removed (*n* = 19; Figure S2). A preliminary principal component analysis (PCA) led to the identification of 11 samples from other species misidentified as grey seals (Figure S3), and analysis in ngsRelateV2 (Hanghøj et al. 2019) resulted in the removal of two closely related or duplicate samples (HG008, HG594) from the dataset (Figure S4). We also followed the ‘perfect‐individual’ approach as described in Orlando et al. (2013) to identify and exclude error‐prone samples from downstream analyses. Briefly, this method assumes equal genetic distances of samples to an outgroup species and the error rate is calculated as an excess/deficit of derived alleles from the outgroup (D05320— *Phoca vitulina* ) compared to a ‘perfect‐individual’. The ‘perfect‐individual’ was sample HG171 (The Sound, Sweden), selected for its high depth of coverage relative to all other samples. This analysis resulted in the removal of two samples (HG559, HG577) with high relative error rates (Figures S5 and S6). Following the above filtering steps, the final dataset consisted of 188 grey seal samples (Table S1).

### 2.6 Genotype Likelihood Calling and Genomic Region Filtering

Genotype likelihood calling and genomic region filtering were conducted independently for the global dataset, for a NE Atlantic‐Baltic Sea dataset (excluding the NW Atlantic), and for the NE Atlantic and Baltic datasets separately. We limited genotype likelihood calling to autosomal chromosomes as reported by DNAZoo. We called genotype likelihoods using ANGSD v.0.94 (Korneliussen et al. 2014) with the SAMtools mode (‐GL 1), considering only positions with a mapping quality score of at least 30 (‐minMap 30), base quality scores of at least 30 (‐minQ 30), a SNP likelihood ratio test *p*‐value of < 10^−6^ (‐SNP_pval 1e‐6), a minor allele frequency of at least 5% (‐minMaf 0.05), data for which at least 75% of the samples (‐minInd), and the remaining default settings. Following genotype likelihood calling, we assessed site inbreeding coefficients and performed a HWE ratio test with PCAngsd v.0.98.2 (Meisner and Albrechtsen 2019), and subsequently removed flagged loci using the ‐hwe_tole 1e‐6 parameter. Depths of coverage for the remaining loci were calculated using ANGSD v.0.94 with the options ‐dumpCounts 1, ‐doDepth 1, and ‐doCounts 1. Loci in the 10th and 90th percentile of the depth distribution were pruned in R v.4.1.2 (R Core Team 2021). These steps resulted in final global and NE Atlantic‐Baltic Sea datasets of 3812 and 3825 SNPs, respectively, which were used for all subsequent analyses.

### 2.7 Population Clustering and Evolutionary Relationships

Population structure was assessed using the filtered loci for each dataset with a principal components analysis (PCA) implemented in PCangsd v.0.98.2. In addition, we performed an admixture analysis for the global dataset with NGSadmix (Skotte et al. 2013). For this analysis, we simulated the number of ancestral populations (*K*) from 2 to 17, representing the total number of sample localities. For each value of *K*, we performed 100 simulations to ensure model convergence, defined as the top three maximum likelihood results falling within two log‐likelihood units. The NGSadmix analysis was subsequently visualised with pong (Behr et al. 2016). To assess the performance of values of *K*, we calculated the residuals under models that converged using EVALADMIX (Garcia‐Erill and Albrechtsen 2020).

The evolutionary relationship among individual grey seals was inferred by calculating a distance matrix in ngsDist v.1.0.10 (Vieira et al. 2016), which was then used as input for building a neighbour‐joining (NJ) phylogenetic tree in FastMe v.2.1.6.1 (Lefort et al. 2015), and subsequently visualised in R v.4.1.2 (R Core Team 2021). As an outgroup population for both analyses, ddRADseq data from three NW Atlantic harbour seals were included.

### 2.8 Genetic Differentiation, Isolation by Distance (IBD), and Relative Migration Rates

To quantify genetic differentiation, we calculated pairwise *F*_ST_ between all localities using the filtered global dataset. Using ANGSD with default settings, we calculated the site allele frequency likelihood for each population with the ‐doSaf 1 command and calculated the 2d‐SFS for each locality pair with the realSFS command. Finally, we examined putative signatures of isolation by geographic distance as in Gose et al. (2024). Minimum marine distances were calculated using an R script from Assis et al. (2013). *F*_ST_ values were transformed to a continuous scale as genetic distance = *F*_ST_/(1−*F*_ST_) before correlating these to geographic distances. Significance of the correlation between genetic distance and geographic distance was assessed with a redundancy analysis (RDA) and Mantel test implemented and visualised in R.

Relative rates of migration based on allele frequencies were assessed with the function divMigrate (Sundqvist et al. 2016) implemented through the R package diveRsity (Keenan et al. 2013). Kattegat samples were excluded due to a low sample size. Allele frequencies were calculated with ANGSD using the ‐doPost 1, ‐doGeno 4, ‐doCounts and ‐dumpCounts 2 commands. The ANGSD beagle file output was first converted to a VCF with the R‐script angsd2vcf.R (https://github.com/rcristofari/RAD‐Scripts/blob/master/angsd2vcf.R), loaded with vcfR (Knaus and Grünwald 2017), converted into a genid object with adegenet (Jombart 2008), paired with sampling location metadata, and subsequently converted into the genepop format required for divMigrate using graph4lg (Savary et al. 2021). We calculated the effective number of migrants statistic (*N*_m_) (Alcala et al. 2014) using 1000 bootstraps and filtered for *N*_m_ greater than 0.3 to interpret the most meaningful patterns of migration.

### 2.9 Genetic Diversity

To quantify the genetic diversity within each locality, we estimated the proportion of heterozygous sites per individual and averaged these values for each locality using the global SNP dataset. We inferred the site allele frequency likelihood for each sample individually in ANGSD with the ‐doSaf 1 command, including only loci that had a minimum depth of coverage of 10 (‐setMinDepthInd 10). We subsequently calculated the 1d‐SFS with the realSFS command within ANGSD using the default settings. In addition, spatial relationships of genetic diversity metrics including nucleotide diversity (π), allelic richness (*A*_R_), and heterozygosity (*H*_O_) were investigated with the R package wingen (Bishop et al. 2023). The genetic diversity metrics were calculated with the function window_gd(), which calculated each metric along a spatial sliding window with rarefaction to account for differences in sample sizes. The command krig_gd() was used to interpolate diversity metrics for unsampled regions using the kriging method. The output was masked to grey seal suitable habitat as assessed through a species distribution model accessed from AquaMaps (Kaschner et al. 2019).

### 2.10 Recent Demographic History

To infer past changes in effective population size (*N*_e_) we applied the GONE (Santiago et al. 2020) method, which relies on the observed spectrum of linkage disequilibrium (LD) of pairs of loci over a range of recombination rates to infer *N*_e_ within the last 200 generations of a species. Allele frequencies were calculated with ANGSD using the ‐doPost 1, ‐doGeno 4, ‐doCounts and ‐dumpCounts 2 commands. To prepare the GONE input files, the ANGSD beagle file was first converted to a VCF with the R‐script angsd2vcf.R (https://github.com/rcristofari/RAD‐Scripts/blob/master/angsd2vcf.R) and subsequently converted with PLINK into the required .ped and .map input files. We created three data subsets for the NW Atlantic, NE Atlantic and Baltic Sea to investigate demographic history separately, as population structure may impact *N*_e_ estimates. Our parameters for GONE followed the default settings and assumed a constant recombination rate of 1 cM/Mb across the genome. For each regional subset, we ran 40 iterations of GONE, randomly excluding 10% of individuals per run. The mean *N*_e_ and 95% confidence intervals were calculated and visualised in R. Finally, to explore the effect of sample size on *N*_e_ estimates, we performed additional analyses systematically downsampling the Baltic region from 82 to two samples by increments of five.

## 3 Results

### 3.1 Population Clustering and Evolutionary Relationships

We examined patterns of population clustering across the grey seal's range by PCA and NGSadmix analysis. The global PCA analysis revealed three main genetic clusters that correspond to samples from the NW Atlantic (Muskeget Island and Sable Island), NE Atlantic (Iceland, Froan, Kola Peninsula, Isle of May, Skomer Island, Wadden Sea and Kattegat) and Baltic Sea (Belt Sea, The Sound, Ertholmene, Stockholm Archipelago, Gulf of Riga, Gulf of Finland and Bothnian Bay), respectively (Figure 1B; Figure S1 for reference map, Figure S7; Table S2). The first and second principal components accounted for 5.1% and 3.4% of the variation in the dataset, respectively, clustering localities roughly according to their geographic proximity. An additional PCA of NE Atlantic and Baltic Sea grey seals not only confirmed this pattern, but revealed additional finer scale structure with Icelandic grey seals separating from other NE Atlantic animals along PC2 (4.2%; Figure 1C) and Froan (Norway) and Kola Peninsula (Russia) grey seals separating along PC3 (1.6%; Figure S8). The separate clustering of NW Icelandic, SE Icelandic, Norwegian‐Russian and North Sea region grey seals was also evident when the PCA was restricted to NE Atlantic samples with PC1 (3.7%) and PC2 (2.4%) separating Iceland and Froan‐Kola Peninsula, respectively, from other NE Atlantic localities (Figure 1D).

**FIGURE 2:**
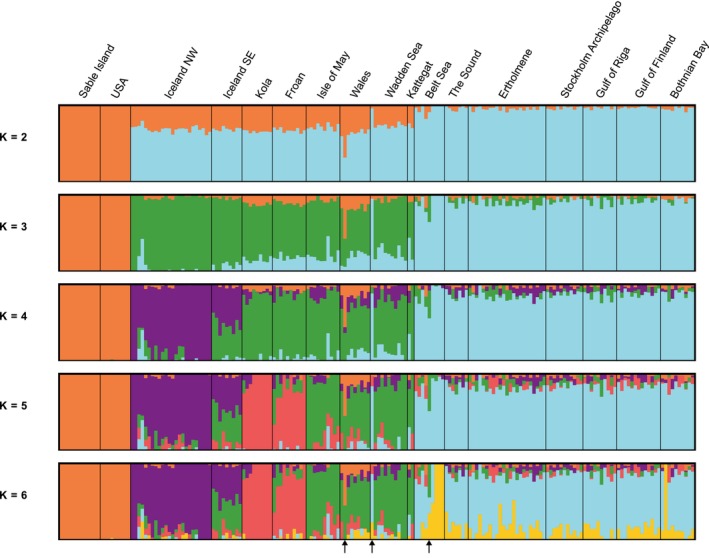
Grey seal population genomic structure assessed by admixture analysis in NGSadmix for K2‐6, see Figure S11 for K6‐17. Arrows below *K* = 6 indicate grey seals that are migrants or admixed individuals between different regional populations.

The NGSadmix analysis pointed to overall similar patterns of population clustering (Figure 2, Figure S11). For *K* = 1–7, the models converged with the top three iterations falling within two log‐likelihood units of each other, while *K* = 8–17 did not converge. EvalAdmix supported *K* values 5, 6 and 7 as having the residuals closest to zero (Figure S10), indicative of the existence of five to seven genetic clusters. In addition to supporting the overall patterns from the PCAs, the NGSadmix analysis provided some additional insights on finer scale substructuring. Specifically, at *K* = 4, grey seals in NW Iceland appeared to form their own genetic cluster, whereas grey seals in SE Iceland were admixed with those in Isle of May (East Scotland), Skomer Island (South Wales), the Wadden Sea and Kattegat. Similarly, at *K* = 5, grey seals from Froan (Norway) and Kola Peninsula (Russia) formed their own genetic cluster, and at *K* = 6, a few animals from the Danish locality of Rødsand in the Belt Sea region carried a unique genetic signature also found in a few other Baltic Sea animals.

Overall, the population clusters revealed by the PCA and NGSadmix analyses were well‐defined, although we note that a few animals appear to be migrants or admixed (Figures 1 and 2). This includes a single animal sampled in the Wadden Sea (HG517), which clustered with Baltic Sea grey seals and thus appears to be a migrant, and a Belt Sea animal (HG301), which showed admixed ancestry between the Baltic and NE Atlantic. Perhaps most strikingly, a grey seal from Skomer Island (HG589) appeared to have equal amounts of NW and NE Atlantic ancestry, and generally most Welsh grey seals appeared to have a faint NW Atlantic genetic signature in the NGSadmix analysis.

The individual‐based NJ phylogenetic analysis placed all NW Atlantic grey seals in a separate clade with > 90% bootstrap support, pointing to their marked genetic distance from all other grey seals (Figure S11). NE Atlantic grey seals tended to form subclades according to sampling locality, with animals from Skomer Island placed as a sister clade to NW Atlantic grey seals, Froan (Norway) and Kola Peninsula (Russia) grey seals forming two sister clades, NW and SE Iceland forming two other sister clades, and grey seals from other NE Atlantic localities being more dispersed in the tree. Baltic Sea animals tended to form their own clade, yet with lower than 90% support, little phylogenetic structure according to sampling locality, and also including a few NE Atlantic grey seals.

### 3.2 Genetic Differentiation, Isolation by Distance and Relative Migration Rates

The estimates of pairwise genetic differentiation (*F*_ST_) supported the population clusters identified by the PCA and NGSadmix analyses, revealing moderate to high levels of genetic differentiation between localities among each of the three main population clusters (Figure S12). Levels of differentiation tended to be higher between NW Atlantic and other grey seal localities (*F*_ST_ = 0.084–0.135) than between NE Atlantic and Baltic Sea localities (*F*_ST_ = 0.034–0.057). Within each region, differentiation was highest among NE Atlantic localities (*F*_ST_ = 0.000–0.044), and non‐existent among NW Atlantic localities and among Baltic Sea localities, perhaps with the exception of the Belt Sea (*F*_ST_ = 0.000–0.006).

**FIGURE 3:**
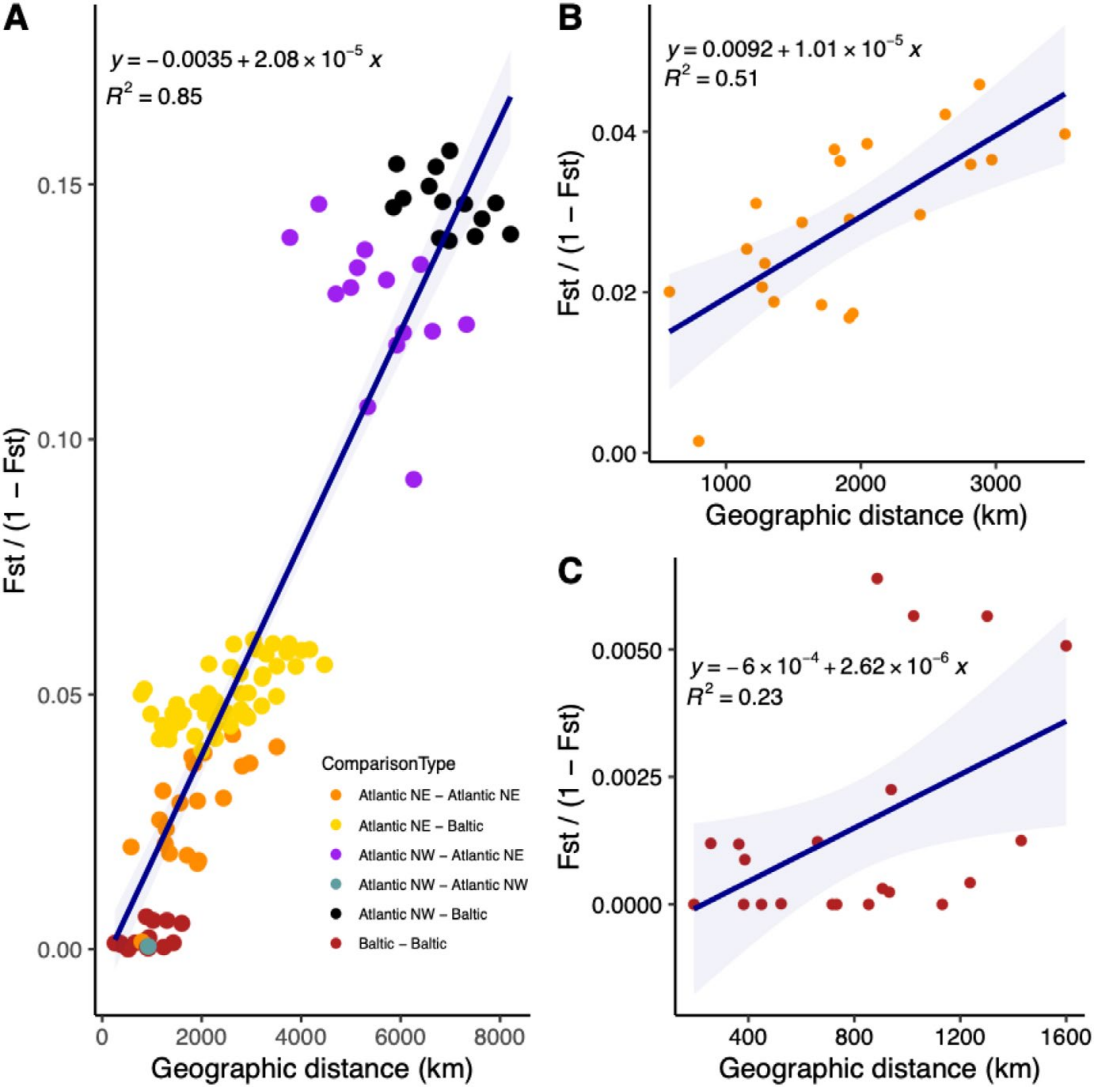
Comparison of standardised *F*_ST_ values with geographical distances between localities to test for isolation by distance across the grey seal's range (A), as well as within the Northeast Atlantic (B) and Baltic Sea (C).

For all sampling sites included in the isolation by distance (IBD) analysis, the RDA and Mantel test showed a strong and statistically significant correlation between genetic distance and at‐sea geographic distance (Figure 3A; RDA: *R* ^2^ = 0.85, *p* = 0.001; Mantel: *r* = 0.83, *p* = 0.001). Pairwise comparisons within the NE Atlantic also exhibited moderate and significant correlations between genetic distance and geographic distance (Figure 3B; RDA: *R* ^2^ = 0.51, *p* = 0.008; Mantel: *r* = 0.38, *p* = 0.001), while the observed correlation within the Baltic Sea (Figure 3C; RDA: *R* ^2^ = 0.24, *p* = 0.064; Mantel: 0.13, *p* = 0.149) appeared driven by comparisons to the Belt Sea, with little other evidence of IBD among the remaining sites in the Baltic Sea. We noted that some locality pairs were outliers in that they are either more (above trend line) or less (below trend line) genetically differentiated than expected given their geographic distance. For example, in the global analysis, Sable Island and Iceland (Atlantic NW—Atlantic NE) were more genetically differentiated than expected, while Muskeget and Skomer Island (Atlantic NW—Atlantic NE) were less differentiated than expected (Figure 3A). Within the NE Atlantic comparisons, Isle of May and Wadden Sea were also less differentiated than expected given their geographical distance (Figure 3B).

**FIGURE 4:**
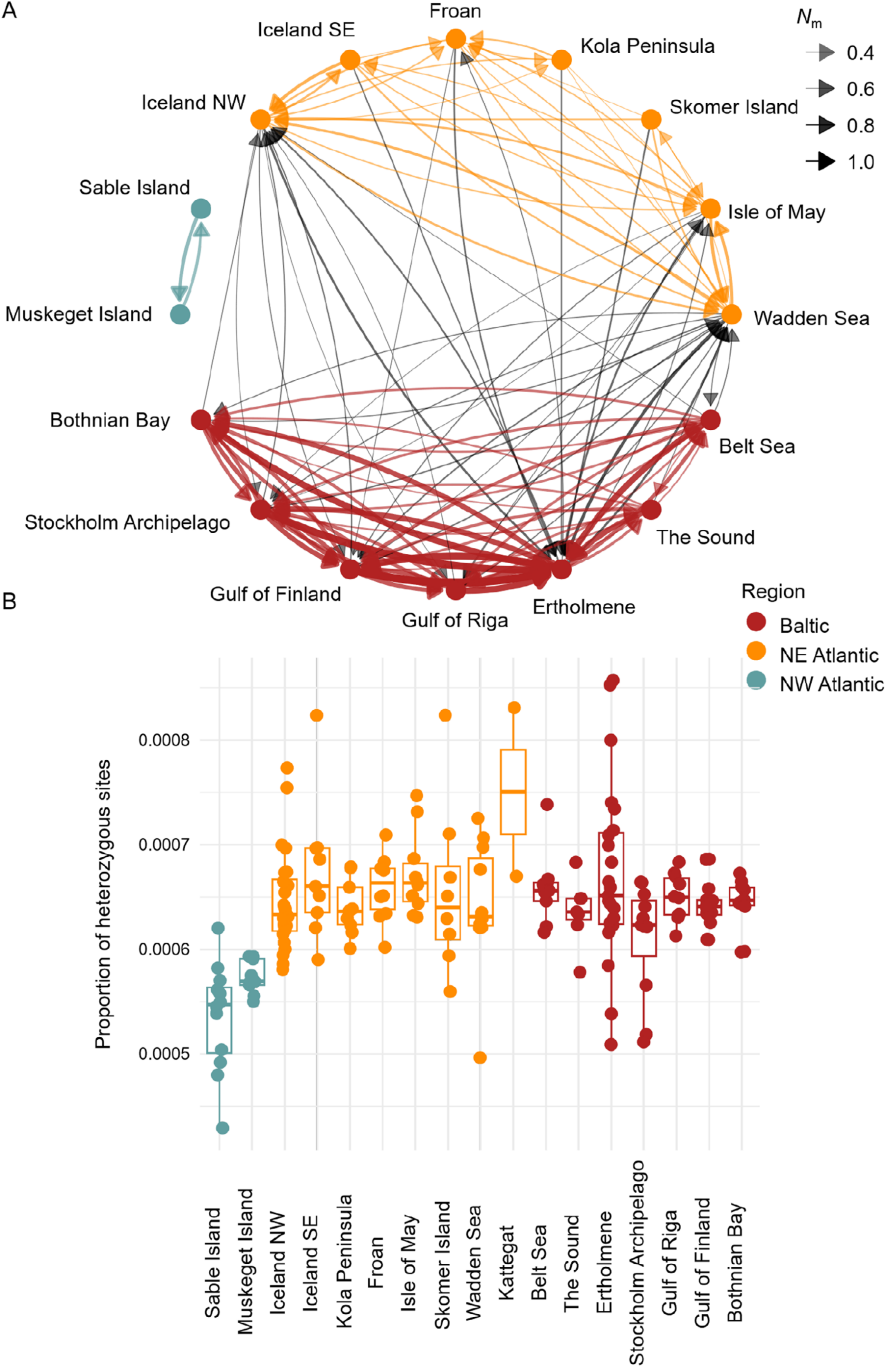
Connectivity and diversity of grey seals inferred from (A) effective migration parameter (*N*_m_) assessed with divMigrate and (B) genetic diversity estimated as the proportion of heterozygous sites per individual. The legend indicating the sampling locality region is applicable to both subplots. The arrow weight legend for *N*_m_ indicates the relative strength of effective migration. Black arrows indicate effective migration between different regions while effective migration within a region is coloured according to the region.

The estimates of relative migration rates among grey seal localities supported the overall clustering of NW Atlantic, NE Atlantic and Baltic localities with substantially higher levels of connectivity within than between the three main regions (Figure 4A; Figure S13). While NW Atlantic grey seals appear highly isolated from those in the NE Atlantic and Baltic Sea, the latter populations appear to exchange some level of migrants; in particular, the Danish Wadden Sea and Ertholmene appear to be inter‐population (subspecies) migration junctions. Finally, we note that the levels of connectivity within the NE Atlantic are lower than within the Baltic, consistent with some population genetic substructure within the NE Atlantic and very little population structure within the Baltic Sea.

### 3.3 Genetic Diversity

Genetic diversity was estimated as the proportion of heterozygous sites per individual and averaged for each locality (Figure 4B). Baltic Sea and NE Atlantic grey seals had overall similar levels of genetic diversity, whereas genetic diversity was substantially lower in the NW Atlantic, indicating that this population may have experienced a strong bottleneck in the past. Few other patterns emerged from the heterozygosity estimates, although we note that grey seals at the Ertholmene locality in the Baltic Sea were characterised by surprisingly large variation in diversity, with some animals having very high levels of heterozygosity and others very low. These observations were supported by spatial nucleotide diversity, allelic richness and heterozygosity estimates calculated in sliding windows with wingen (Figure S14). All three metrics were lowest in the NW Atlantic, further supporting the possibility of a population bottleneck. The Ertholmene locality had relatively high genetic diversity metrics compared to neighbouring populations within the Baltic, also supporting the observations made from the genome‐wide heterozygosity estimates. Since our samples originate from well‐defined sites, the interpolated values across seaways from wingen are not informative of diversity at unsampled sites, as they have been for dispersed species in other studies.

### 3.4 Recent Demographic History

**FIGURE 5:**
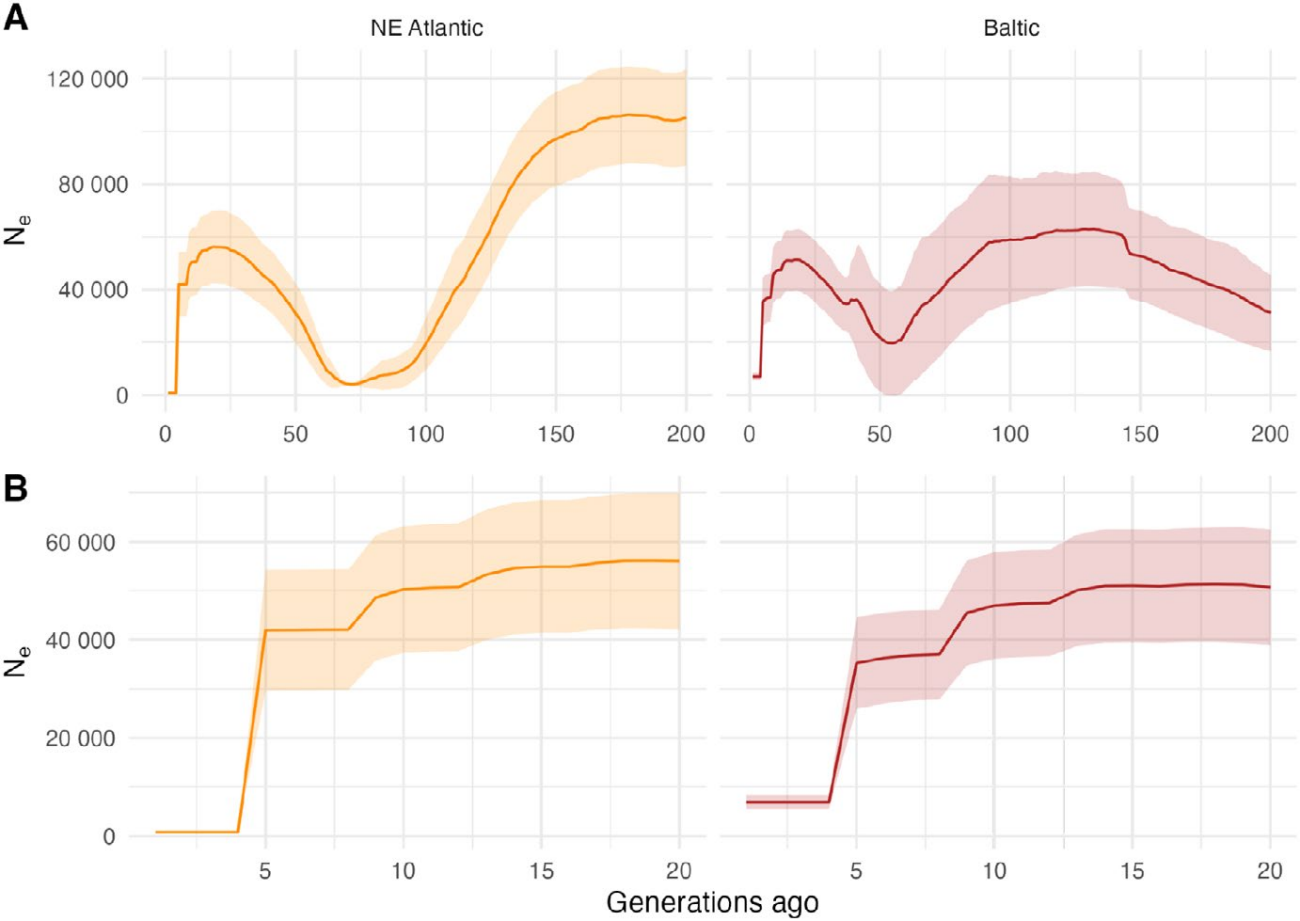
Demographic analyses for the NE Atlantic (orange) and Baltic Sea (red) regions for the past (A) 200 and (B) 20 generations using GONE (Santiago et al. 2020). The NW Atlantic region was excluded from this analysis due to a limited sample size. Forty iterations were run excluding 10% of the population with each iteration to calculate a mean (bolded line) and 95% confidence intervals (shaded ribbons).

The inference of recent effective population size (*N*_e_) changes using LD patterns across the genomes revealed fluctuating *N*_e_ across the NE Atlantic and Baltic Sea (Figure 5). We limited our analysis to the past 200 generations, as the GONE method performs most reliably for this time period (Santiago et al. 2020). Iterative down‐sampling of the number of seals included in the analysis showed inflated *N*_e_ estimates at low sample sizes (Figure S15) and we therefore refrained from including the NW Atlantic in this analysis due to its comparatively small sample size. The NE Atlantic experienced a gradual decline in *N*_e_ starting 150 generations ago, being at its lowest 90–60 generations ago and then increasing until 10 generations ago, where the population again declined to a present day *N*_e_ ~ 820 (95% CI: 785–855). The Baltic Sea *N*_e_ gradually increased from 200 to 125 generations ago, and then was stable for 25 generations before it began gradually declining to a low 50 generations ago, after which it increased or remained stable until a stark drop 5 generations ago to a present *N*_e_ ~6938 (95% CI: 5508–8367).

## 4 Discussion

Global grey seal population structure, metapopulation dynamics and recent demographic history in relation to human impacts have remained elusive. Here, we present the first range‐ and genome‐wide analysis of grey seal population structure and diversity, which supports the existence of three main grey seal populations in the NW Atlantic, NE Atlantic and Baltic Sea, respectively, and reconciles previously inconsistent findings of fine‐scale structure within regions. By broadening the geographic and molecular scope of data collection, we can infer ecological contexts within and across regions that support connectivity or suggest cryptic barriers to dispersal. We also draw upon these data to elucidate the recent evolutionary history of the species and consider the impacts of historical demography on contemporary genomic diversity. Finally, we consider the value of a range‐wide analysis to inform management and conservation of a wide‐ranging species distributed across international boundaries.

### 4.1 Range‐Wide Inference Reveals Population Substructure and Local Metapopulation Dynamics

Consistent with the main population boundaries indicated in earlier studies, our range‐wide analysis identified three main genetic clusters that correspond to grey seal samples from the NW Atlantic, NE Atlantic, and Baltic Sea. As predicted by geographic distance, but incongruent with current subspecies designations, genetic differentiation was greatest between the NW Atlantic cluster and the two eastern clusters. Though separated by less geographic distance, significant genetic differentiation between the NE Atlantic and Baltic Sea clusters is consistent with patterns of separation between these regions observed in several other marine species, including marine mammals, fish and invertebrates (e.g., Johannesson and Andre 2006; Wenne et al. 2020; Autenrieth et al. 2024). These consistent observations across diverse species point to either past and/or contemporary barriers to dispersal, perhaps caused by the Baltic Sea's semi‐enclosed physical nature and/or distinct environmental characteristics of low salinity and periodic sea‐ice coverage that drive local adaptations and site fidelity.

Within each of the three main genetic clusters, levels of regional sub‐structure and migration between sites varied greatly. In the NE Atlantic, we present evidence for the existence of hitherto unrecognised genetic subpopulations in northwest Iceland and Norway–Russia, and perhaps SE Iceland, but report little to no genetic differentiation in the wider North Sea region, including Isle of May and the Wadden Sea, contrasting previous findings of fine‐scale structuring (Allen et al. 1995; Pomeroy et al. 2000; Gaggiotti et al. 2002). The deviation from previous studies might owe to study design, but could also reflect a change in metapopulation dynamics with an increase in connectivity among sites driven by population recovery and recruitment (Russell et al. 2019). We found little to no genetic differentiation and generally higher levels of migration among Baltic Sea localities (except perhaps for the Belt Sea), contrasting studies by Karlsson et al. (2005) and Graves et al. (2009), who reported strong site‐fidelity and significant genetic differentiation among Baltic Sea grey seals, respectively. Finally, we detected no genetic structure within the two Northwest Atlantic sites (Muskeget and Sable Islands), consistent with Cammen, Schultz, et al. (2018) and Wood et al. (2011).

Our genomic observations are generally consistent with the wide range of movements observed from tagging data among haul‐out sites, particularly in the North Sea (Brasseur et al. 2014; Peschko et al. 2020; Carter et al. 2022), within the Baltic Sea (van Beest et al. 2022, 2019; Oksanen et al. 2014) and within the NW Atlantic (Goulet et al. 2001; Breed et al. 2006; Nowak et al. 2020, 2023). Taken together, these results indicate that, while grey seals may exhibit fidelity to their natal breeding area, the level of dispersal to other sites can be sufficient to maintain genetic connectivity at wide spatial scales. In particular, such connectivity appears to be driven by source populations at high densities facilitating immigration and net recruitment above intrinsic growth rates in sink populations at lower densities (Dietz et al. 2003, 2015; McConnell et al. 2012; Kyhn, Dietz, et al. 2024; Kyhn, Galatius, et al. 2024; Russell et al. 2019; Thomas et al. 2019).

An examination of cases with lower than expected genetic differentiation or higher relative migration rates from across the species range provides insights into the ecological contexts that support connectivity in the face of ecological tendencies for differentiation. Within regions, higher relative migration rates and lower than expected differentiation between sites likely reflect metapopulation source‐sink dynamics. Similar dynamics have recently been described for harbour seals in the UK associated with regional declines (Carroll et al. 2020). Yet, in grey seals, these dynamics appear to be occurring at a larger geographical scale than described for harbour seals and associated with recent population growth rather than decline. As grey seal populations recover following historical hunting and associated range contractions, dispersal from remaining breeding sites will increase as they reach carrying capacity and emigration rates grow. In our study, the lower than expected genetic differentiation between Isle of May (East Scotland) and the Wadden Sea likely results from the latter being recolonised from Scottish populations (Russell et al. 2019), and similar dynamics have been proposed to explain the lack of genetic differentiation between Muskeget Island and Sable Island populations in the Northwest Atlantic (Cammen, Schultz, et al. 2018; Wood et al. 2020). Although these sites appear to violate expectations of genetic structure resulting from natal site philopatry, it is possible that the historical local extinction and more recent recolonisation of grey seal localities in these areas and others have erased past signatures of genetic differentiation. Indeed, ancient and historical DNA studies of Baltic Sea and NW Atlantic grey seals have suggested such population replacements and shifts driven by prehistoric and historic local extinctions and subsequent recolonisation (Ahlgren et al. 2022; Fietz et al. 2016; Cammen, Vincze, et al. 2018).

Beyond connectivity within regions, range‐wide population genomic data also facilitates detection of dispersal between regions, which is of particular relevance for species with the ability to undertake long‐distance movements, like the grey seal (McConnell et al. 1999; Brasseur et al. 2015; Carter et al. 2022; Nowak et al. 2023). We observed a single Welsh animal with mixed NW Atlantic and NE Atlantic ancestry, which could be the result of a long‐distance dispersal (and successful mating) event. The observation of a Belt Sea individual of mixed NE Atlantic and Baltic ancestry and high relative migration rates at nearby Ertholmene may also reflect connectivity between genetically distinct regions, in this case separated by distinct breeding habitat and season and perhaps periodic historic isolation rather than geographic distance. This signal of reproductive admixture is consistent with recent observations of grey seals in the NE Atlantic and Baltic Sea increasingly overlapping in distribution, with intermediate localities serving as resting sites (Ertholmene) and/or resting and breeding sites (Danish Wadden Sea and Belt Sea) (Fietz et al. 2016; Galatius et al. 2020; Sveegaard et al. 2024). This highlights the need to continue genetic monitoring, particularly in boundary regions where genetic, morphological and behavioural differences may in time take the form of a continuous gradient between the two regions.

### 4.2 Historical Population Declines and Recent Recovery

In the NE Atlantic and the Baltic Sea, the demographic analyses revealed repeated fluctuations in *N*_e_ within the past 200 generations, yet did not detect the very recent increase in population size, possibly because of the rapid rate of recovery. Assuming a grey seal generation time of 12 years, calculated as the mean age of reproducing females (Jonasson et al. 2022; Carroll et al. 2024), we infer that NE Atlantic grey seals experienced a 1000‐year bottleneck approximately 200–1300 AD, followed by a 600‐year period of gradual recovery and a more recent period of decline starting approximately 1900 AD. The demographic history of Baltic Sea grey seals is more complex and associated with larger uncertainty, but it appears to have been slightly increasing in *N*_e_ up to 800 AD followed by a 600‐year bottleneck until 1400 AD, a subsequent period of stability and a more recent decline also starting approximately 1900 AD. The inferred recent decline observed in both regions fits surprisingly well with the initiation in the late 19th and early 20th century of large‐scale grey (and harbour) seal culling programmes across Europe to minimise seal‐fishery conflicts, as well as later pollution with environmental toxins, leading to population crashes and local extinctions in both NE Atlantic and Baltic Sea grey seals (Harding and Härkönen 1999; Härkönen et al. 2007; Hauksson 2007; Duck and Thompson 2013; Fietz et al. 2016; Olsen et al. 2018). The dramatic decline in the *N*_e_ of the NE Atlantic grey seal approximately 200–1300 AD is a novel finding, but nevertheless supported by accounts that grey seals had disappeared as a breeding species across most of the North Sea region by the end of the Middle Ages 1500 ad due to hunting and human‐induced habitat alterations (Summers 1978; Reijnders et al. 1995; Lambert 2002; Härkönen et al. 2007). Curiously, a recent study hints at the high value of seal skins in medieval Europe and the possible existence of long‐distance trade networks from Scandinavia and the northern British Isles to supply demanding markets on the continent (Lévêque et al. 2025).

Though small sample sizes in the NW Atlantic precluded parallel analysis of recent demographic history as inferred from population genomic data, our findings of low diversity in NW Atlantic grey seals support previous findings that they also experienced a strong bottleneck, either in recent historic time due to overexploitation, local extinctions and recolonisation from a small number of founding populations (Cammen, Vincze, et al. 2018), or perhaps as a founder event in prehistory (see below). Remarkably, NW Atlantic grey seals have the largest contemporary population size at approximately 400,000 animals (DFO 2022; Wood et al. 2022) compared to NE Atlantic and Baltic Sea currently estimated at approximately 180,000 and 60,000 animals, respectively (NAMMCO 2023; HELCOM 2023). The NW Atlantic grey seals exhibiting low post‐bottleneck diversity and rapid growth are similar to observations in northern elephant seals ( *Mirounga angustirostris* ) and several otariid species bouncing back from the brink of extinction (Hoelzel et al. 2024; Hoffman et al. 2024; Stoffel et al. 2018).

While subject to comparably high hunting pressure and multiple local extinctions (e.g., Härkönen et al. 2007; Hauksson 2007; Fietz et al. 2016; Galatius et al. 2020), NE Atlantic and Baltic Sea grey seals have retained higher levels of diversity than those in the NW Atlantic. As discussed above, the movement capacity of grey seals likely results in metapopulation dynamics operating at large within‐region spatial scales, which, together with the occasional inter‐population migrant, may drive genetic homogeneity and the retention of overall genetic diversity in the NE Atlantic and Baltic Sea. Such metapopulation dynamics have also been proposed for other species subject to historical population declines and recent recovery, such as harbour seals in the United Kingdom (Carroll et al. 2020) and Saimaa ringed seals (*Pusa saimensis*) (Löytynoja et al. 2023). Finally, we note that the *N*_e_/*N*_c_ ratio (effective population size/census population size) observed in the Baltic Sea (6938/60,000 = 11.56%) is substantially higher than that in the NE Atlantic (820/180,000 = 0.46%), and likely that of the NW Atlantic (which could not be estimated). This may result from Baltic grey seals having a less polygynous mating strategy and higher reproductive success (low pup mortality), at least when breeding on ice (Jüssi et al. 2008).

### 4.3 Possible European Origin of the Grey Seal

The phylogenetic analysis points to a European origin of the grey seal with an early split between grey seals currently inhabiting the Baltic Sea and the North Sea, followed by expansion along three main axes north to Iceland, northeast along Norway up to its range‐limit in the Barents Sea region of Russia, and west via Wales to the NW Atlantic (USA and Canada), respectively. Given the glacial history of the region, these splits may have occurred in refugia outside of the species' current range, as has also been proposed for other pinnipeds (Olsen et al. 2025). Of note, the stepping‐stone hypothesis of historical cross‐Atlantic dispersal proposed for harbour seals (Liu et al. 2022) is not supported by our genetic data for grey seals. In fact, pairwise comparisons between sites in the NW and NE Atlantic appear to exhibit a reverse IBD pattern, and the marked genetic differentiation between grey seals in Iceland and USA–Canada indicates that dispersal between these two locations is very rare. Instead, it is possible, given the movement capacity of grey seals, that they might be able to make an Atlantic crossing, without the need for intermediate ice‐ or land‐based resting platforms, as suggested by a single Welsh animal with mixed NW Atlantic and NE Atlantic ancestry. Additional analyses of whole‐genome sequences may be able to further resolve the evolutionary history of the species, including the timing and trajectories of past dispersal events.

### 4.4 Implications for Management of Grey Seals and Other Wide‐Ranging Species

Expanding species sampling from national or regional scales to a range‐wide focus is important for properly delineating MUs, resolving taxonomic delineations, and implementing recovery plans for widely distributed species. In the case of the grey seal, our range‐wide analysis suggests the existence of at least six grey seal genetic units, classified as (1) NW Atlantic, including USA and Canada; (2) Iceland, including all sites from northwest to southeast; (3) Norway‐Russia, although the precise boundary to the North Sea is currently ill‐defined; (4) the North Sea region, from Shetland in northern Scotland to Southeast England, France, Belgium, the entire Wadden Sea region, Limfjorden in northwest Denmark, Skagerrak, Kattegat and possibly also southwestern Norway; (5) Wales, and possibly west England, Ireland and west Scotland, pending additional movement, demographic and genetic data; and (6) the Baltic Sea, including the Danish Belts and possibly southern Kattegat. These units reflect genetic homogeneity resulting from a shared recent evolutionary history, which is one important factor to consider when managing for putative adaptive capacity of a species. However, in the case of grey seals, we note that these do not represent fully isolated genetic units, and vice versa, that some do not constitute fully panmictic (mixing) populations as they are comprised of multiple breeding colonies, some of which currently operate as demographically independent (e.g., the Gulf of St. Lawrence and Sable Island in Canada), further complicating decisions about the scale of management.

Furthermore, we acknowledge that some of our samples were obtained outside of the breeding season and may not represent local genetic breeding populations. Indeed, grey seals are partial migrants (Russell et al. 2013), and thus for management purposes both their genetic and ecological units should be considered. Therefore, the actual scale of management decisions may be finer than the described genetic units, and will likely depend on several other intrinsic (biological) and extrinsic (non‐biological) factors (Smith‐Hicks and Morrison 2021).

We note that there is little evidence that Baltic Sea grey seals are more genetically (this study) or morphologically (Galatius et al. 2022) unique than either of the two major population clusters in the NW and NE Atlantic, respectively. Furthermore, although the Baltic grey seal has a preference for breeding on sea‐ice that is unique in the eastern portion of the species range, it is capable of breeding on land, and grey seals in the NW Atlantic may also breed on ice at some sites (Tinker et al. 1995). In fact, the observed breeding habitat preferences and temporal gap between the pupping seasons of Baltic (February–March) and NE Atlantic (September–December) grey seals, used as a rationale for their designation as separate subspecies, likely result from the historical local extinction of grey seals at intermediate sites in inner Danish waters and the northern Wadden Sea, where pupping occurred in January–February (Reijnders et al. 1995; Harkonen et al. 2007; Olsen et al. 2018; Galatius et al. 2024). As grey seals reestablish these historical breeding and resting sites, we expect a gradual increase in overlap and connectivity between NE Atlantic and Baltic grey seals (Fietz et al. 2016; Galatius et al. 2024), implying that any genetic, morphological and behavioural differences may in time take the form of a continuous gradient rather than a gap between the two subspecies. In contrast, the NW Atlantic seals are considerably more genetically distinct from the NE Atlantic and Baltic Sea seals (*F*_ST_ > 0.1), and the assignment and ordination analyses cluster them without any overlap or sign of admixture (with the possible exception of the admixed Welsh animal). In other northern seals (Phocinae), such genetic distinctiveness has merited the classification of subspecies or ESU/DPS (Löytynoja et al. 2023; McCarthy et al. 2025), supporting a reconsideration of NW Atlantic grey seal classification, including analyses of whole nuclear genomes.

## 5 Conclusions and Perspectives

Mobile or migratory species that transcend national boundaries benefit from international collaboration that fully encompasses a species range to the greatest extent possible. Several global and regional organisations exist to facilitate such cooperation for international nature conservation and management, including the Convention on the Conservation of Migratory Species of Wild Animals (CMS), the Convention on Arctic Flora and Fauna (CAFF), the North Atlantic Marine Mammal Commission (NAMMCO), and the Helsinki Commission (HELCOM). Yet, as a result of resource limitations (e.g., funding, time, logistics) and national priorities, scientific studies are often limited in geographic scope to a subsection of a species' range. We hope that our study provides inspiration for global and regional scientific collaboration to facilitate the most comprehensive species management plans.

As demonstrated here for grey seals, genomic studies at a range‐wide scale can offer new insights into past and present population dynamics, supporting species management into the future. Acknowledging that our data provide a reduced representation of the nuclear genome, there is likely even more to be learned through the generation and analysis of full nuclear genome data from across the grey seal range, including filling in remaining sample gaps. Future whole‐genome studies will enable further high‐resolution inquiries into the species' demographic history, divergence times and putative local adaptations. Combining full genome data with high resolution tagging data may also provide information on the intricacy of metapopulation source‐sink dynamics underlying the recent recovery of grey seal populations and recolonisation of former haul‐outs observed throughout much of the species' range. A deeper understanding of these processes will shed further light on the ecological and genetic contexts that can contribute to conservation success, as well as the consequences of recovery on genomic diversity. For species like the grey seal, experiencing recent and rapid changes in population size and distribution, it may be necessary to regularly revisit subspecies, ESU and MU classifications. Ideally, this includes a comprehensive review of range‐wide whole‐genome and morphology data, along with life history and behavioural traits, which can also show high levels of variation that warrant consideration in conservation and management.

## Author Contributions

K.M.C., S.M.G. and M.T.O. conceived the research; M.L.M., K.M.C., S.M.G. and M.T.O. designed the research; K.M.C., S.M.G., A.G., R.D., J.T., D.J.F.R., M.T.O. provided funding; K.M.C., S.M.G., A.G., R.D., J.T., C.B.T., M.V., M.K., B.M.J., M.P.A., W.D.B., W.B.P., J.A.R., D.J.F.R. and M.T.O. provided materials; K.M.C. and M.T.O. performed the laboratory work; M.L.M. and S.K. analysed the data; M.L.M., K.M.C. and M.T.O. drafted and revised the manuscript; all authors provided editorial inputs and approved the final version of the manuscript.

## Conflicts of Interest

The authors declare no conflicts of interest.

## Supporting information


Data S1

## Data Availability

WGS data are available at the European Nucleotide Archive (Project, PRJEB83329). Scripts used to analyse the data are available at https://github.com/Morgan‐McCarthy/McCarthy‐et‐al‐2025‐Grey‐seals‐ddRAD.
