# Supplementary material for: Range‐Wide Genomic Analysis Reveals Regional and Meta‐Population Dynamics of Decline and Recovery in the Grey Seal
Source: Mol Ecol. 2025 Jun 19;34(14):e17824. doi: 10.1111/mec.17824 (PMC12237086; doi:10.1111/mec.17824)
Supplement: Supplementary file 1 — Data S1 [file MEC-34-e17824-s001.docx]

**Supplemental Information for:**

**Range-wide genomic analysis reveals regional and meta-population dynamics of decline and recovery in the grey seal**

Morgan L. McCarthy^*#^, Kristina M. Cammen^*#^, Sandra M. Granquist, Rune Dietz, Jonas Teilmann, Charlotte Bie Thøstesen, Simon Kjeldgaard, Mia Valtonen, Mervi Kunnasranta, Bjørn Munro Jenssen, Markus P. Ahola ,Britt-Marie Bäcklin, W. Don Bowen, Wendy B. Puryear, Jonathan A. Runstadler, Debbie JF. Russell, Anders Galatius and Morten Tange Olsen^*^

#Equal contribution

*Corresponding authors

**Supplementary Figure S1:** Map displaying sampling locations, select waterways and country names related to the text.


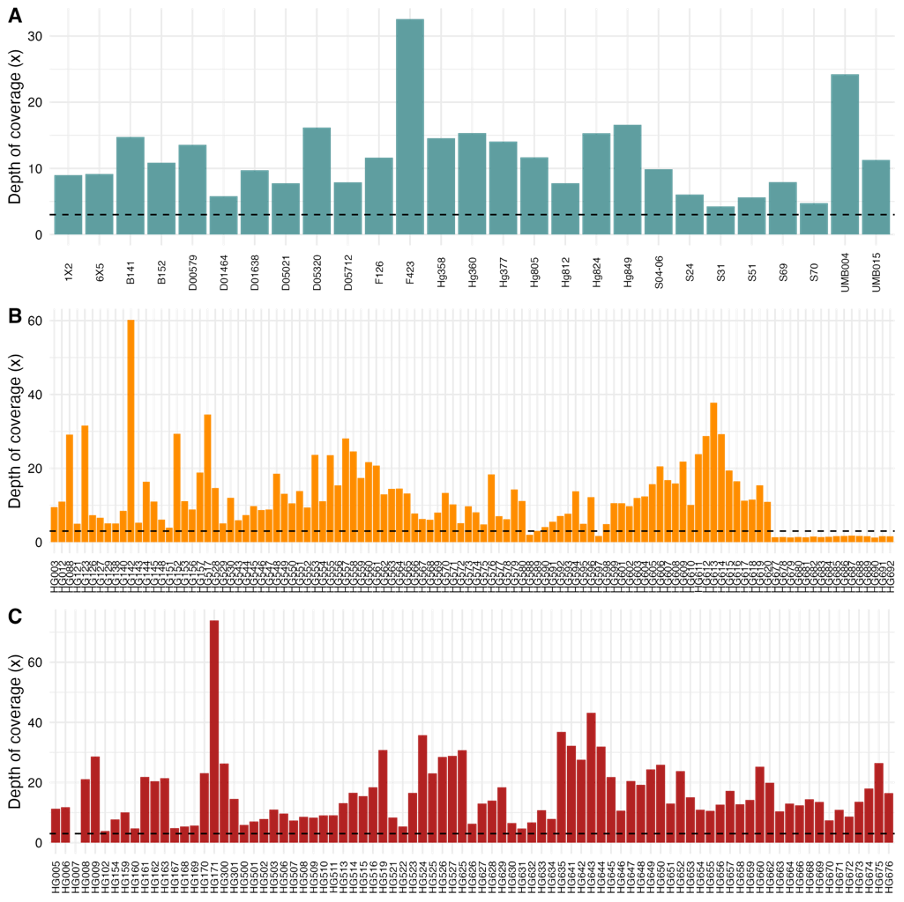


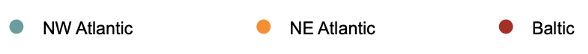


**Supplementary Figure S2**: To eliminate samples that performed poorly during sequencing, we calculated average depth of coverage per sample and filtered samples with an average depth of coverage <3x (samples falling below the horizontal dashed black line). The plot is subdivided into A) NW Atlantic samples, B) NE Atlantic samples, and C) Baltic samples. The entire population from the Faroe Islands falling on the right side of panel B was removed due to low coverage.


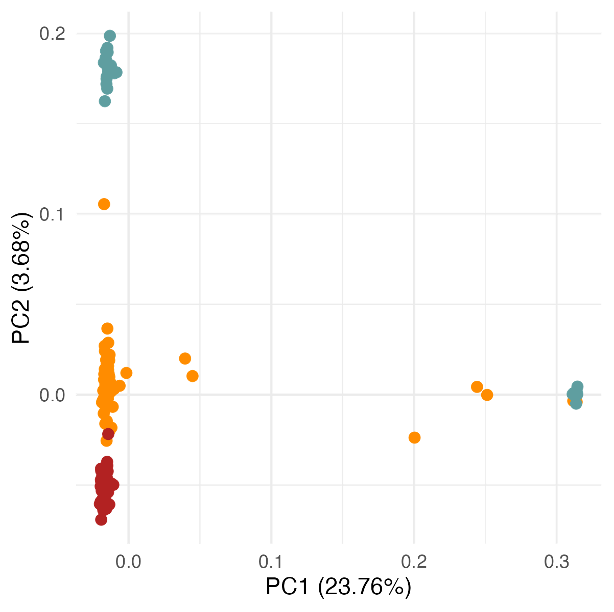


​​**Supplementary Figure S3**: Preliminary PCA to investigate species IDs for sequenced samples. Points greater than zero on PC1 were identified as mislabeled or incorrectly IDed as grey seals and were removed from downstream analyses (n=11).


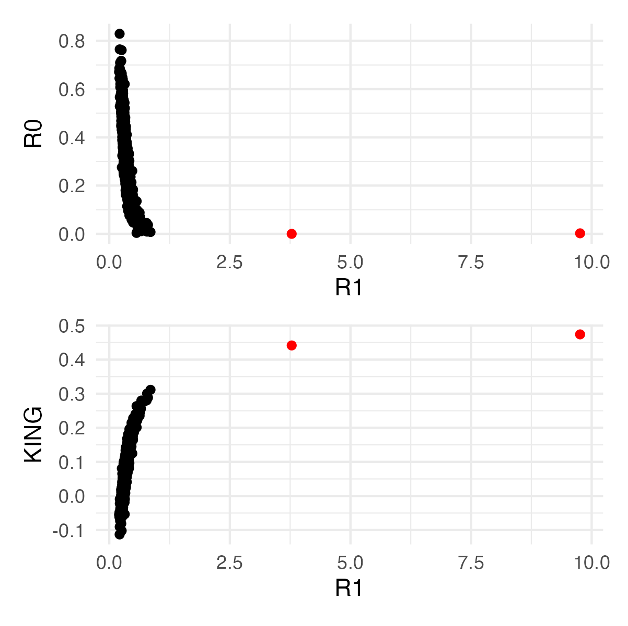


**Supplementary Figure S4:** Plotted results from ngsRelate with suspected related or duplicate samples coloured in red. The two duplicates were (HG008-HG154) and (HG594-HG596). For each pair, the sample with the lower depth of coverage was removed for downstream analyses.


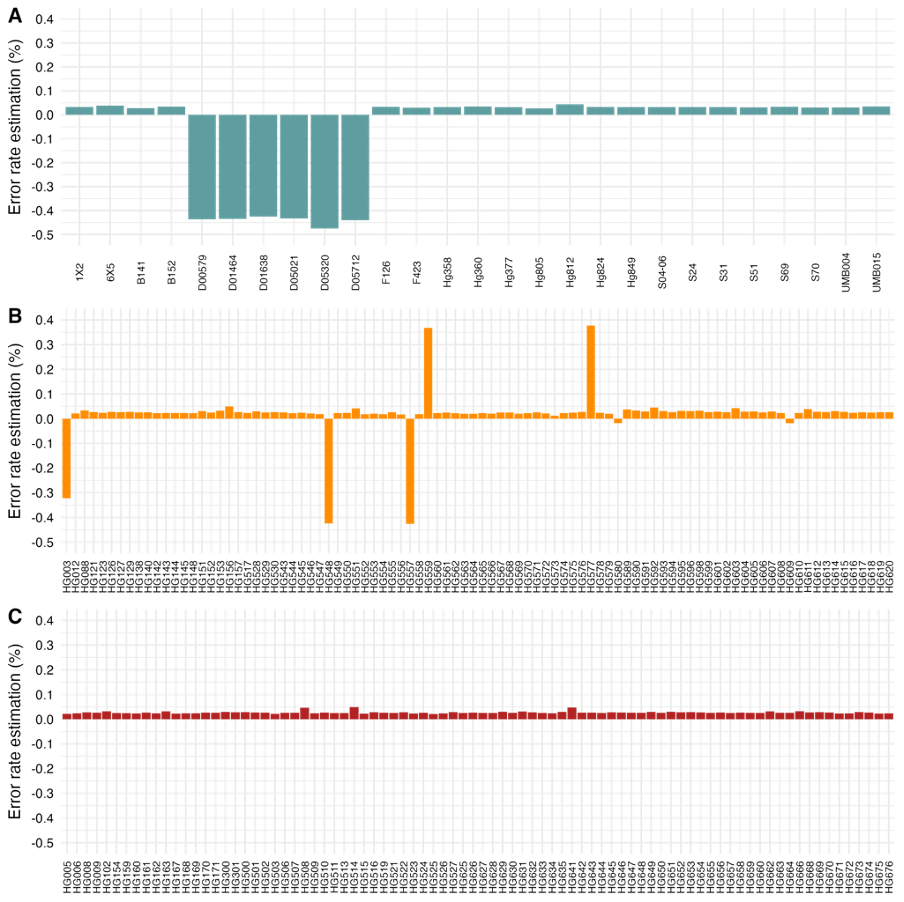


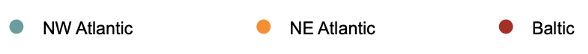


**Supplementary Figure S5**: To determine the relative error rate in the preliminary dataset, we followed the “perfect-individual” approach and plotted the error rate for all samples sequenced with a depth of coverage greater than 3x. The plot is subdivided into A) NW Atlantic samples, B) NE Atlantic samples, and C) Baltic samples. Samples with relative error rates >0.3 and <-0.3 were identical to those identified as mislabeled via initial PCA (Supplementary Figure S3) and were removed from subsequent analyses (n = 11).


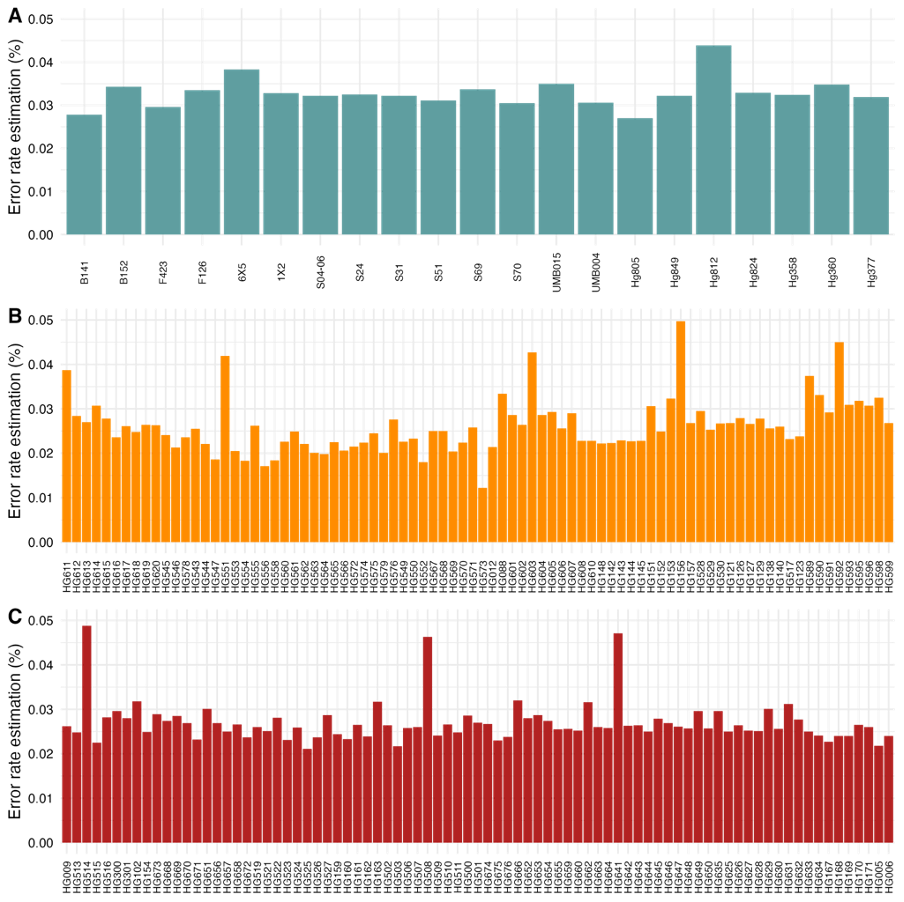


**Supplementary Figure S6**: To determine the relative error rate in the final dataset, we followed the “perfect individual” approach and plotted the error rate for the samples retained in the analyses. The plot is subdivided into A) NW Atlantic samples, B) NE Atlantic samples, and C) Baltic samples.


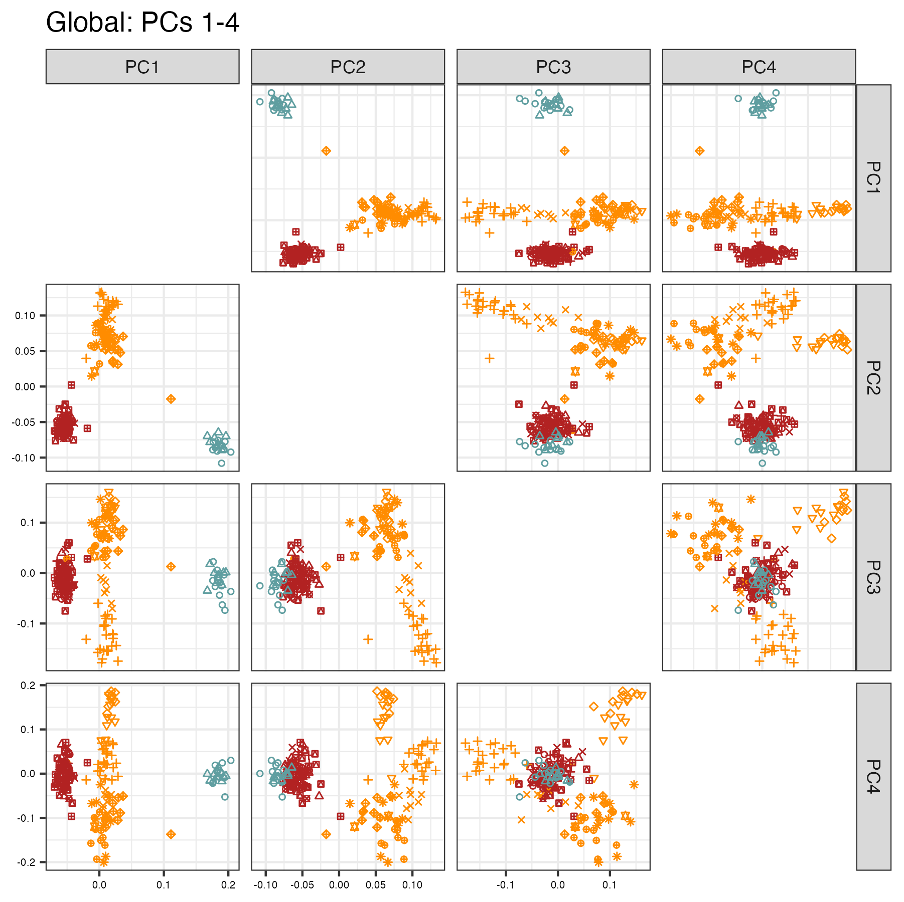


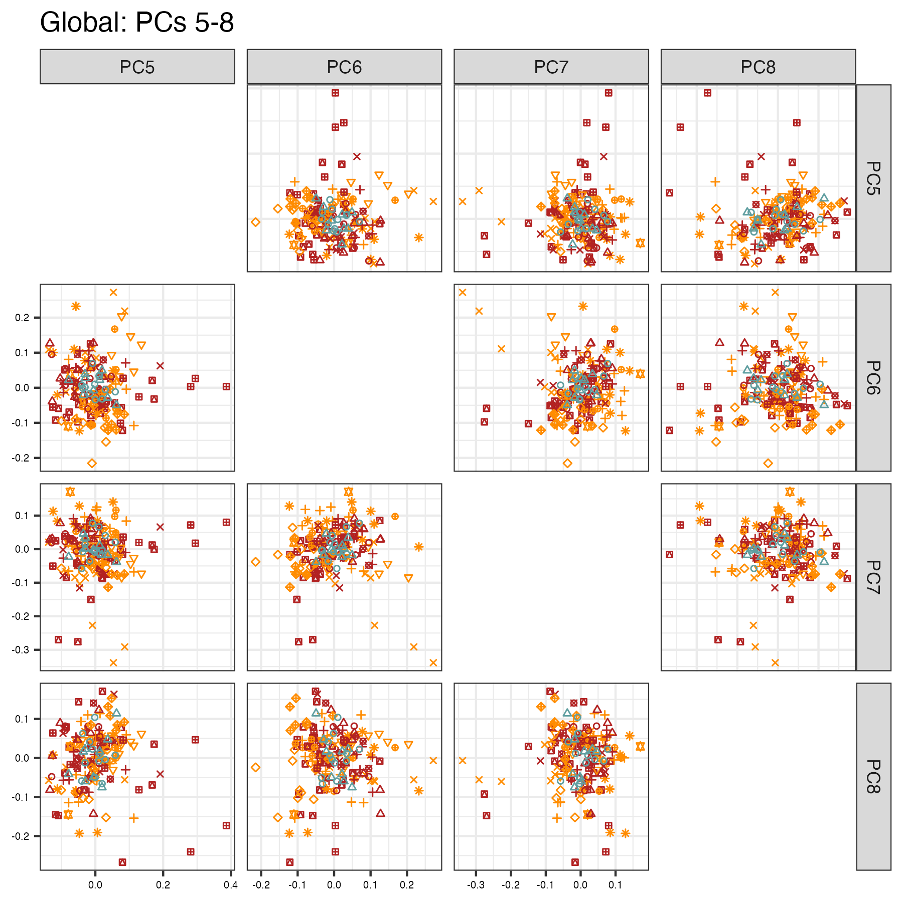


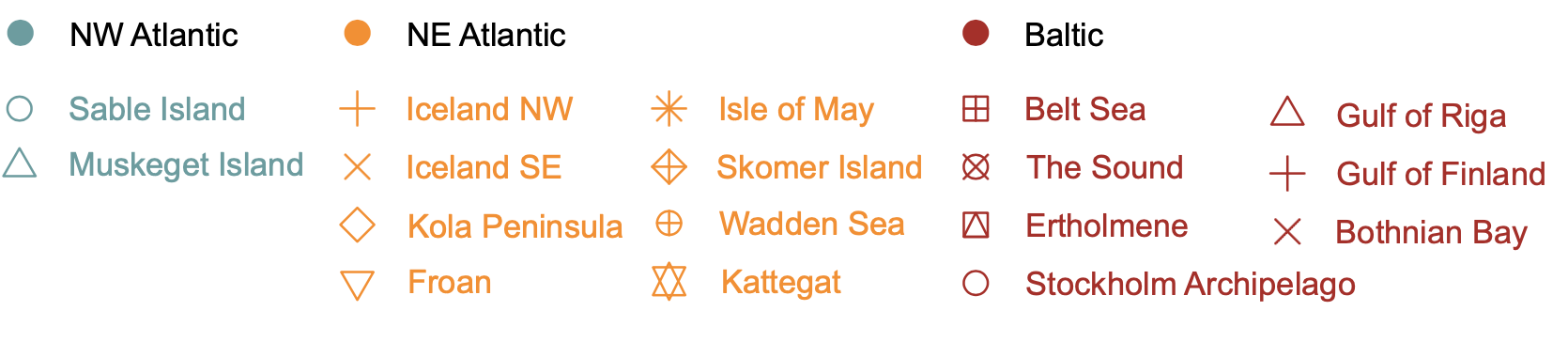


**Supplementary Figure S7**: Principal components 1-4 (top panel) and 5-8 (bottom panel) for the global dataset.


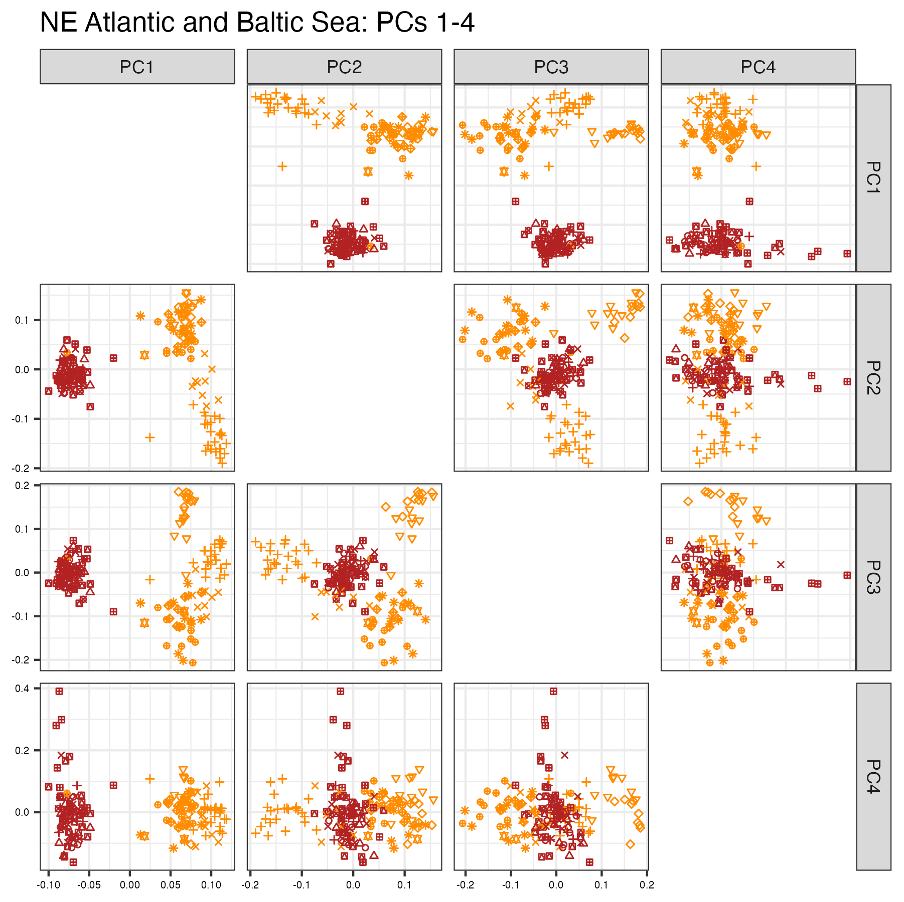


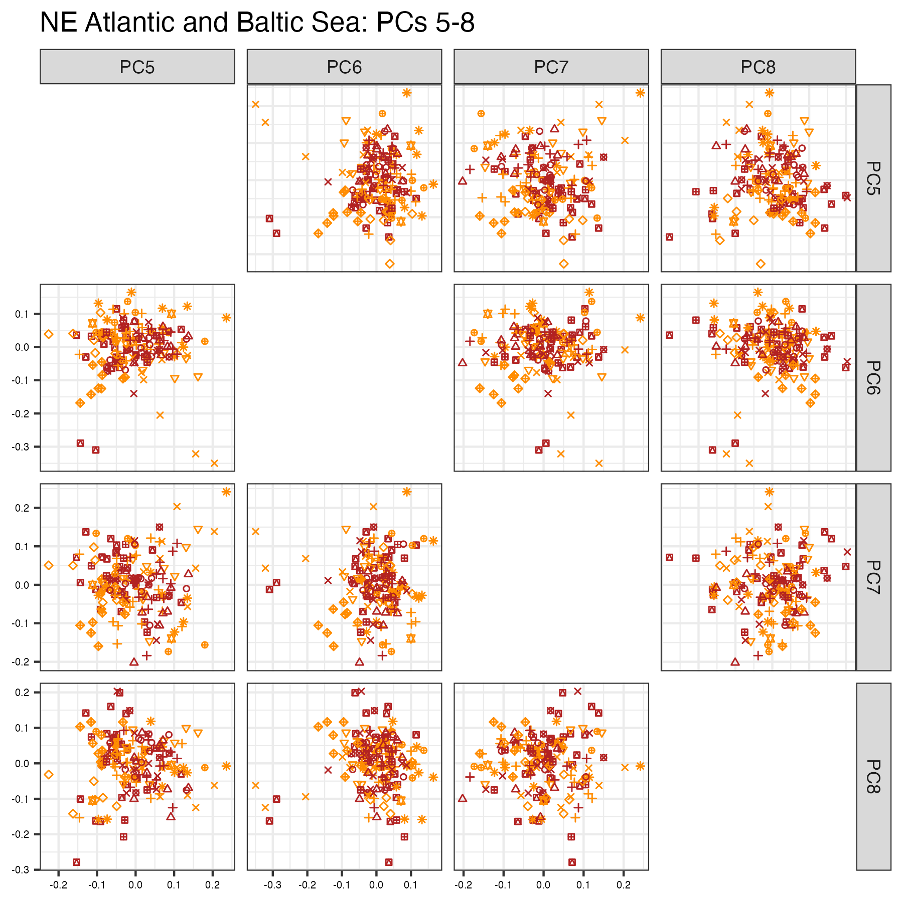


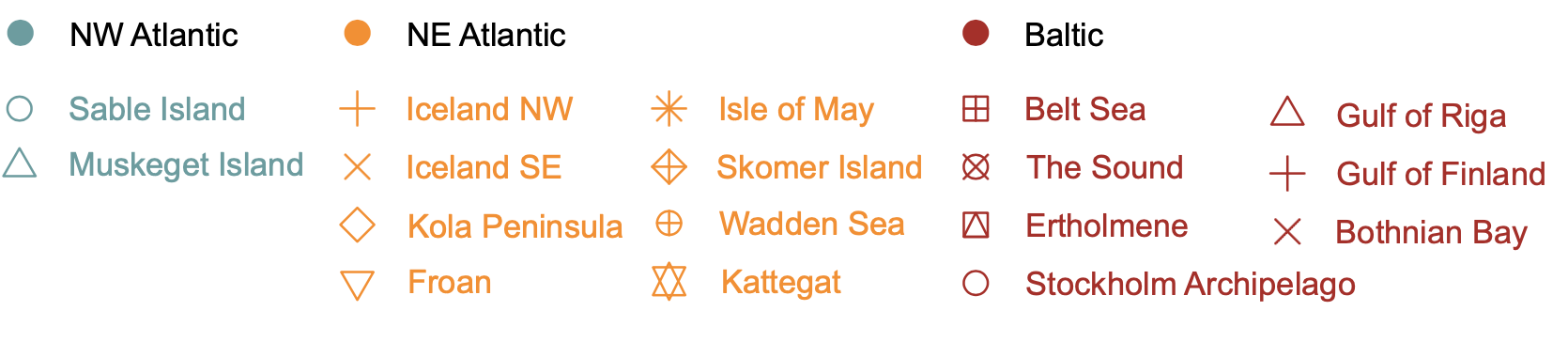


**Supplementary Figure S8**: Principal components 1-4 (top panel) and 5-8 (bottom panel) for the NE Atlantic and Baltic Sea dataset.


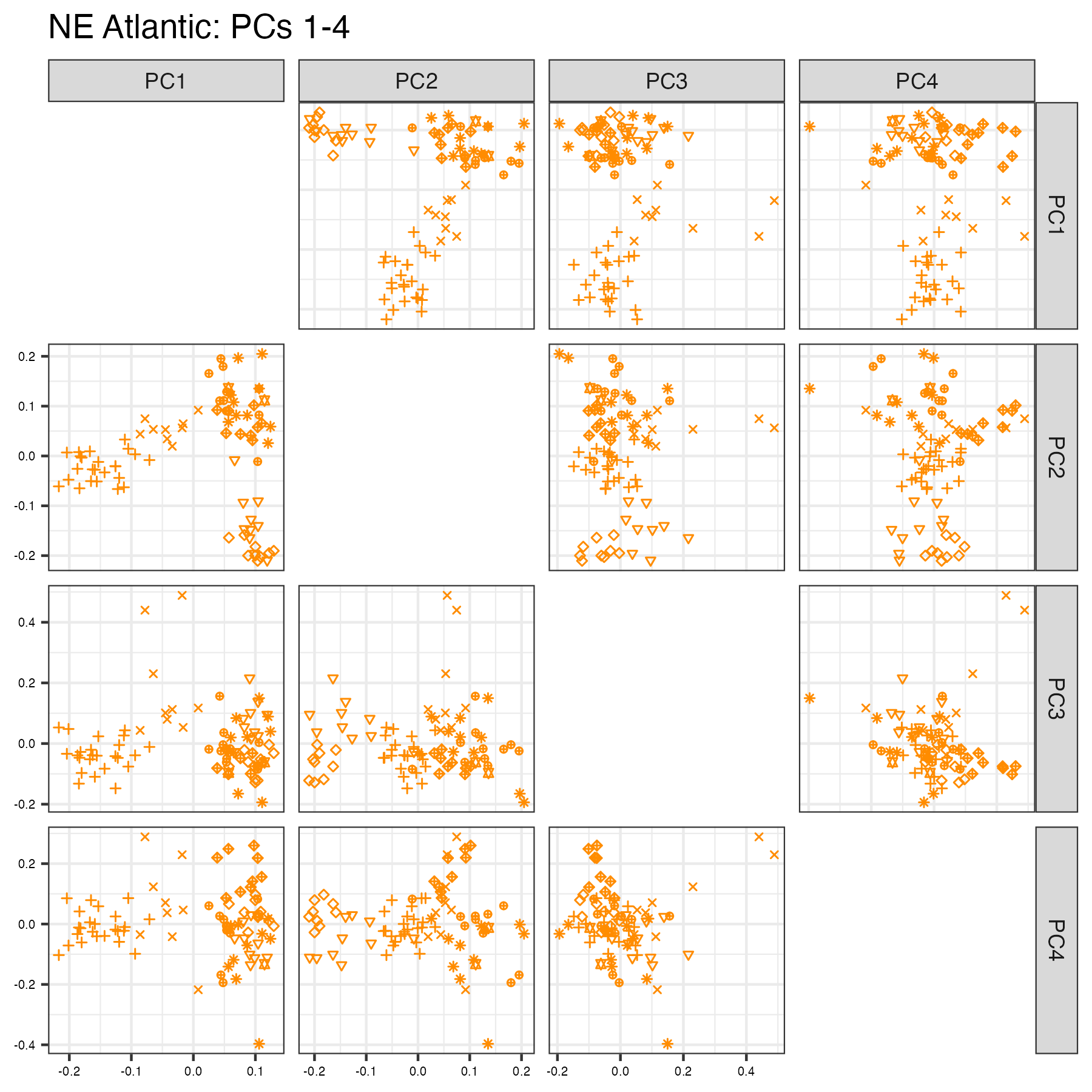


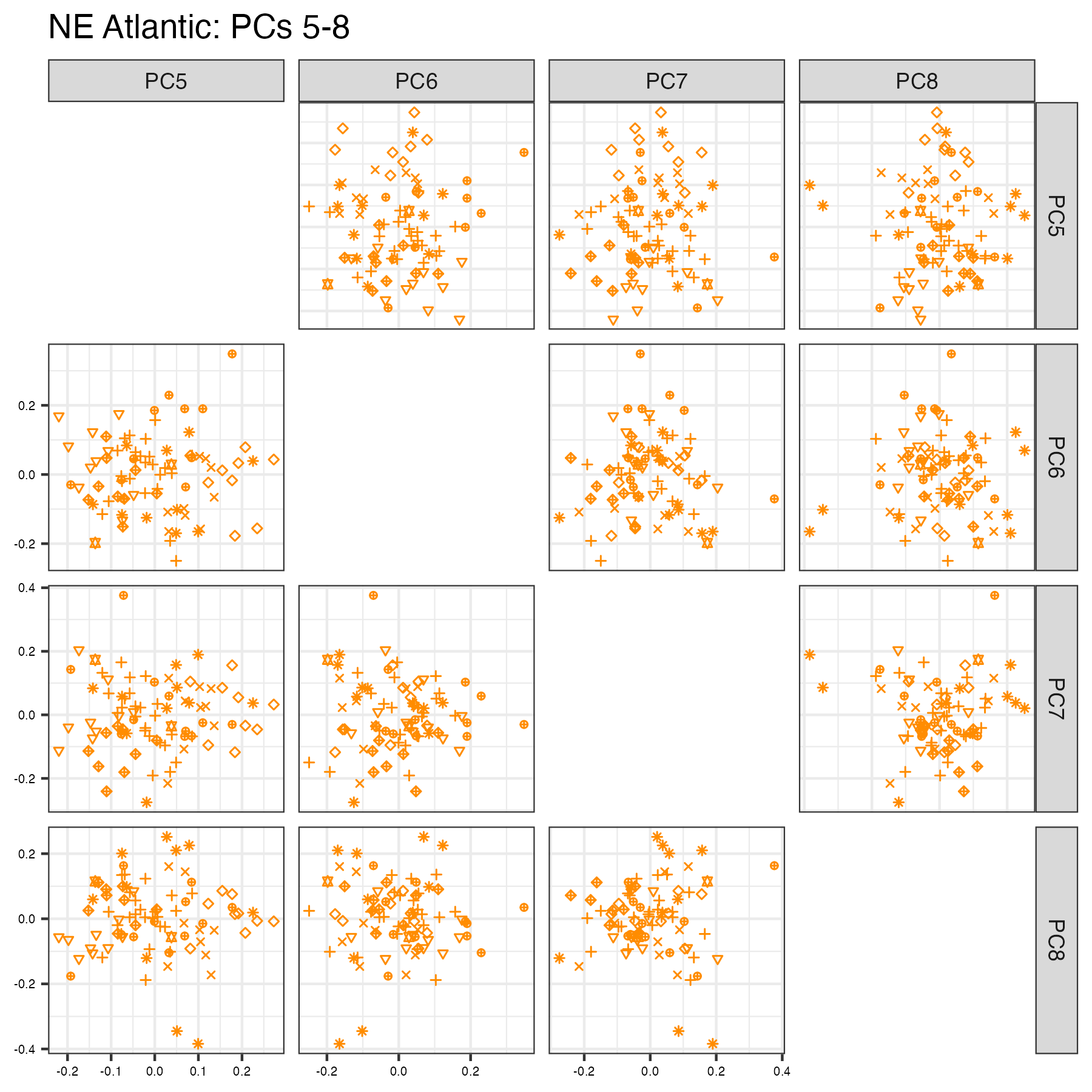


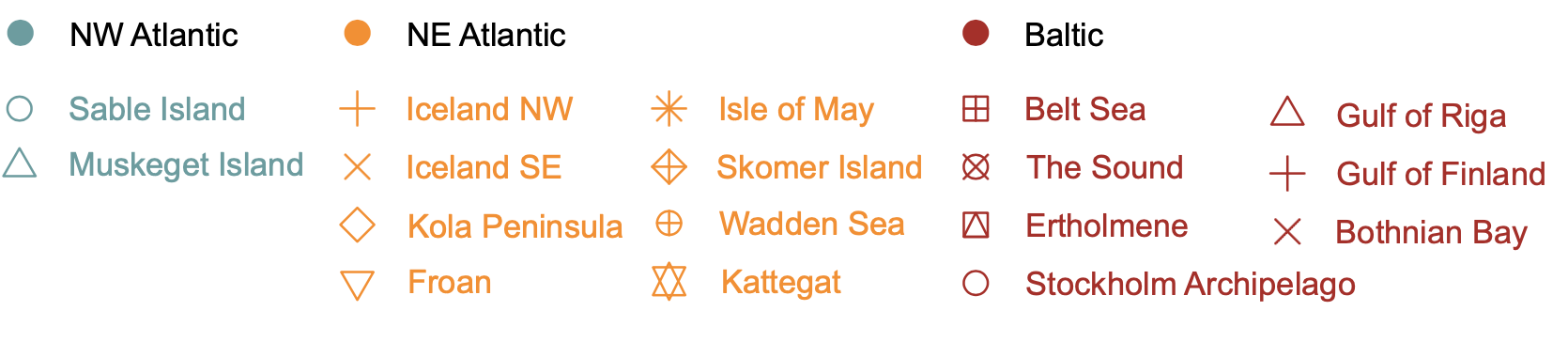


**Supplementary Figure S9**: Principal components 1-4 (top panel) and 5-8 (bottom panel) for the NE Atlantic dataset.

**
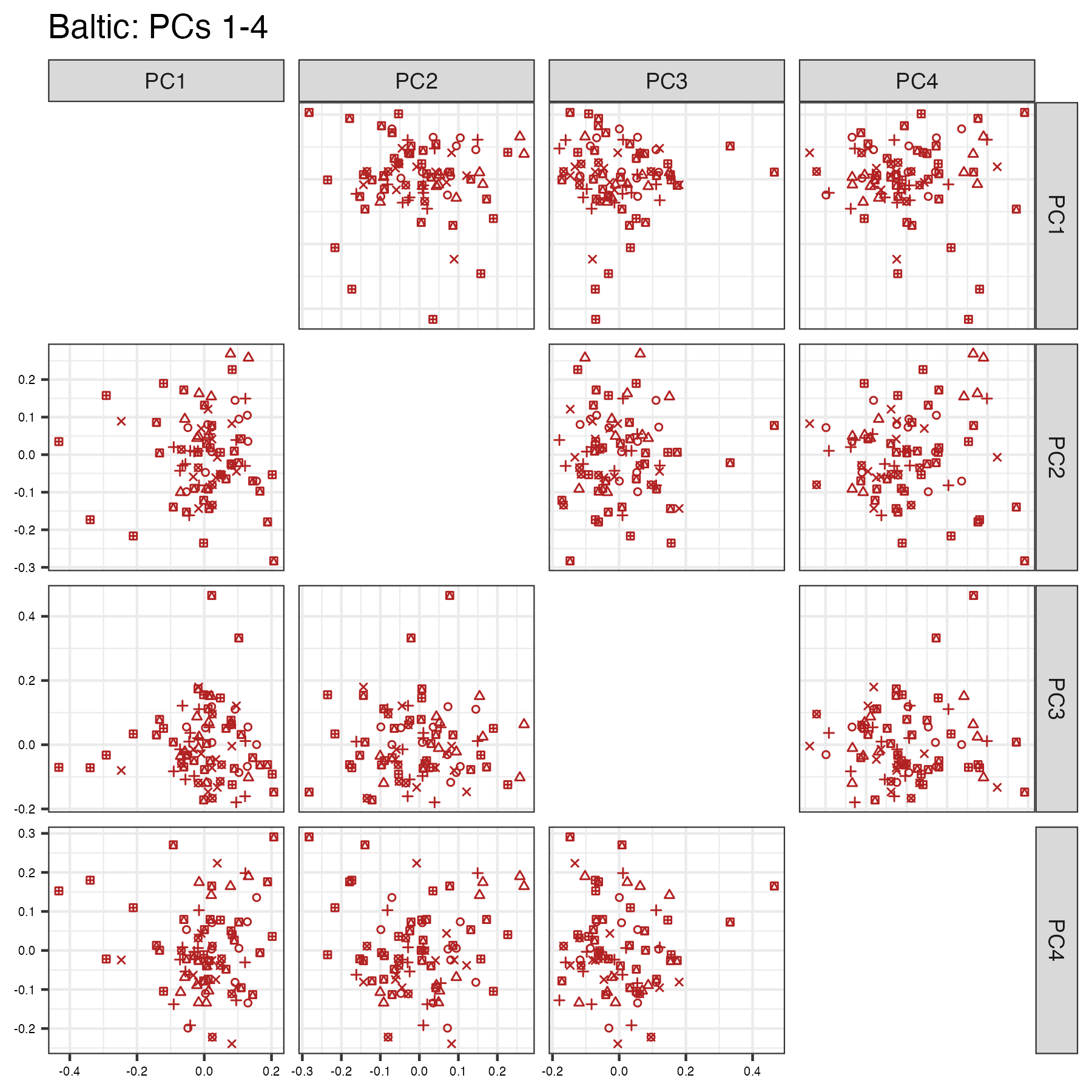
**

**
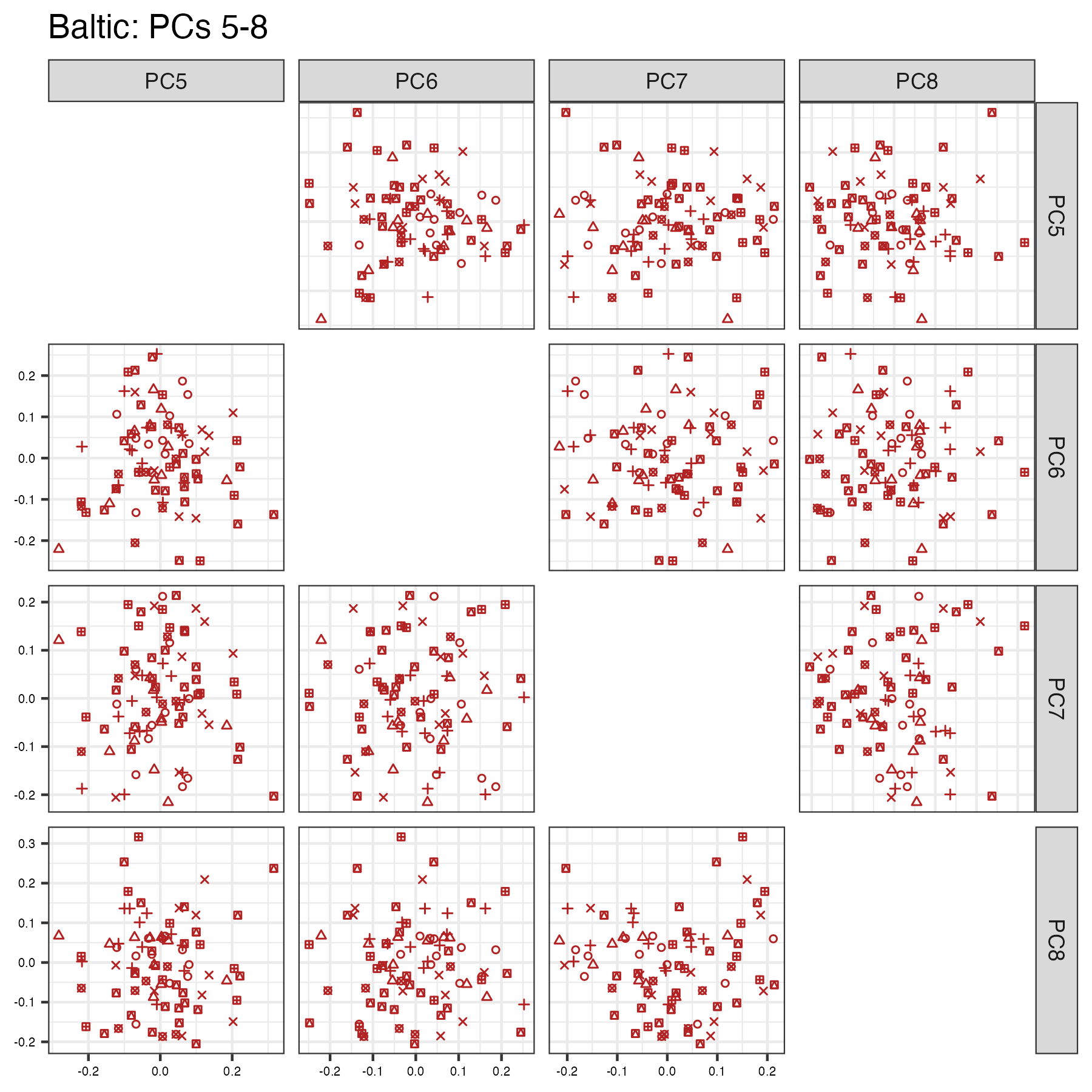
**


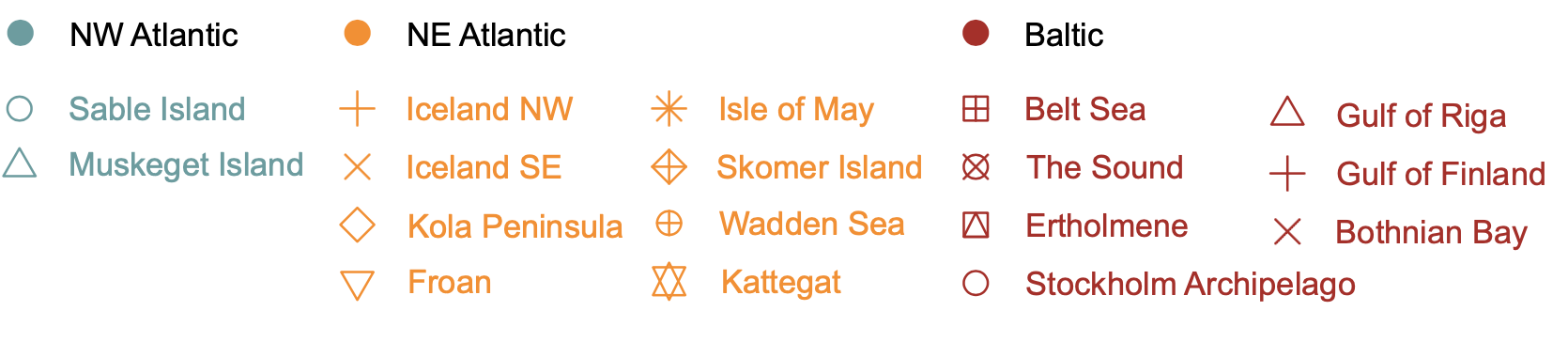


**Supplementary Figure S10**: Principal components 1-4 (top panel) and 5-8 (bottom panel) for the Baltic dataset.

**Supplementary Figure S11:** Grey seal population genomic structure assessed by admixture analysis for K7-17, see Figure 2 for K1-6.


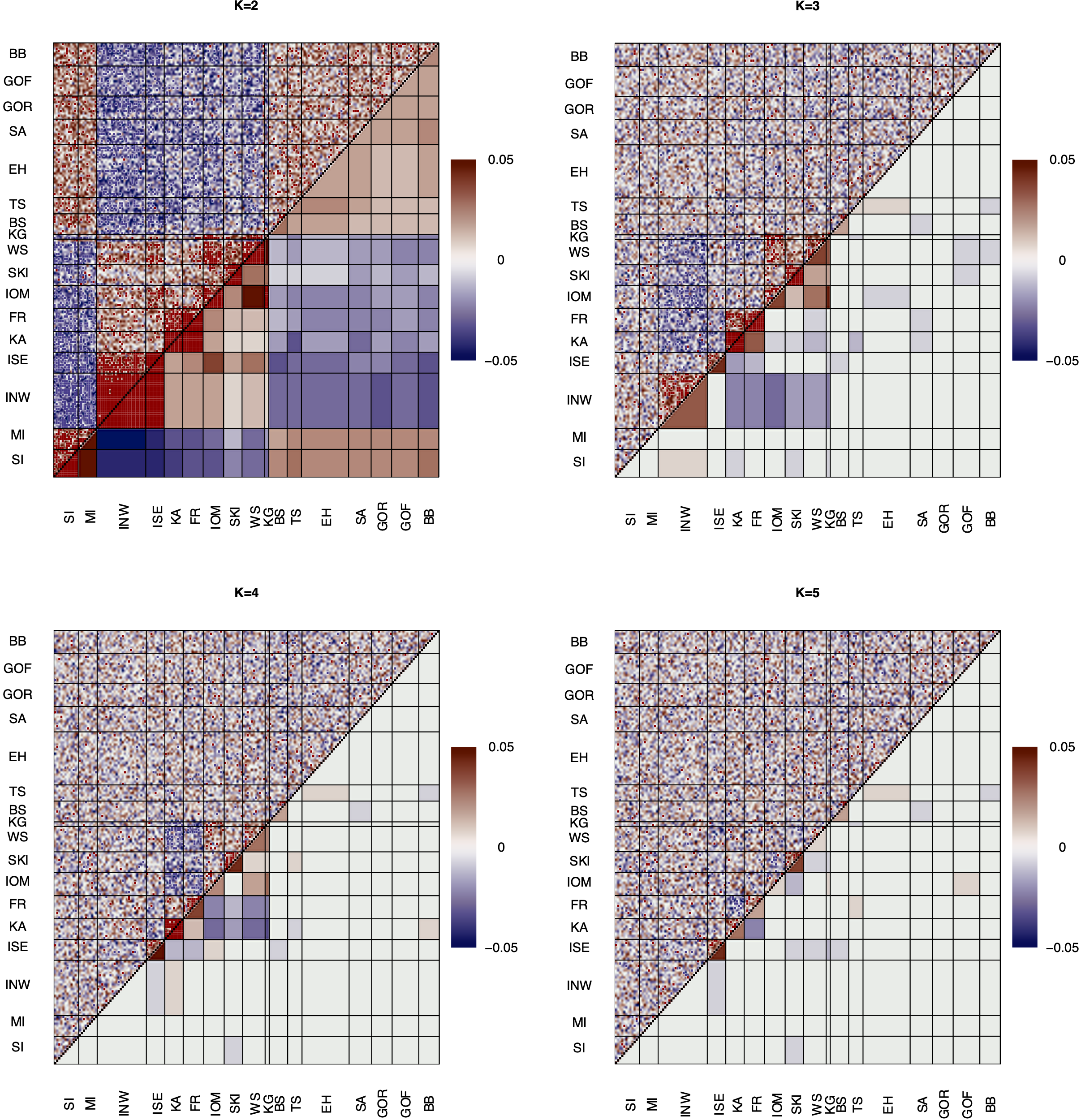


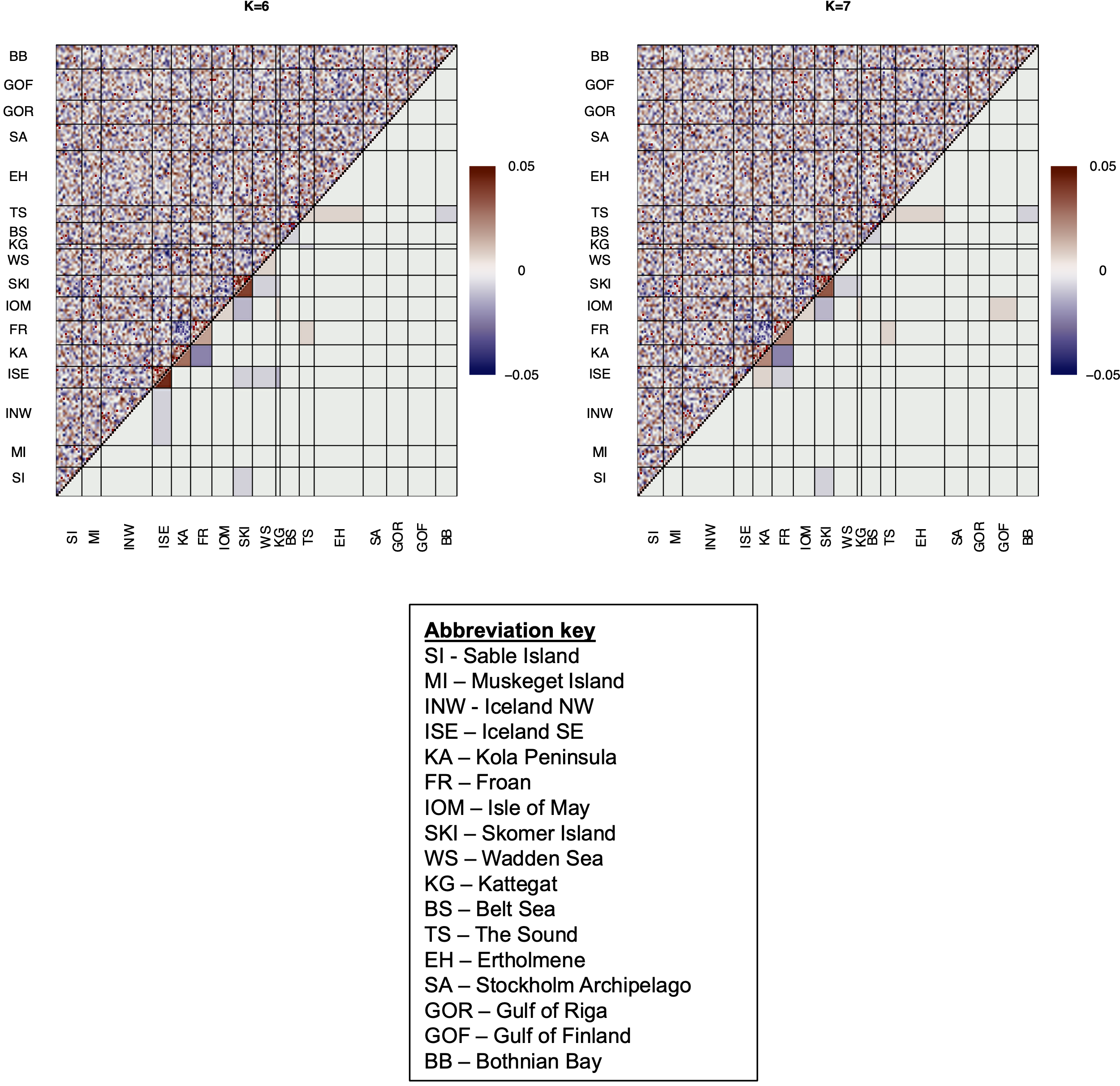


**Supplementary Figure S12:** Output from evalAdmix for values of K that reached convergence (Ks 2-7). The correlation will be close to 0 in the case of a good fit of the data to the admixture model. Plots for K=5, K=6, and K=7 had the best fit.


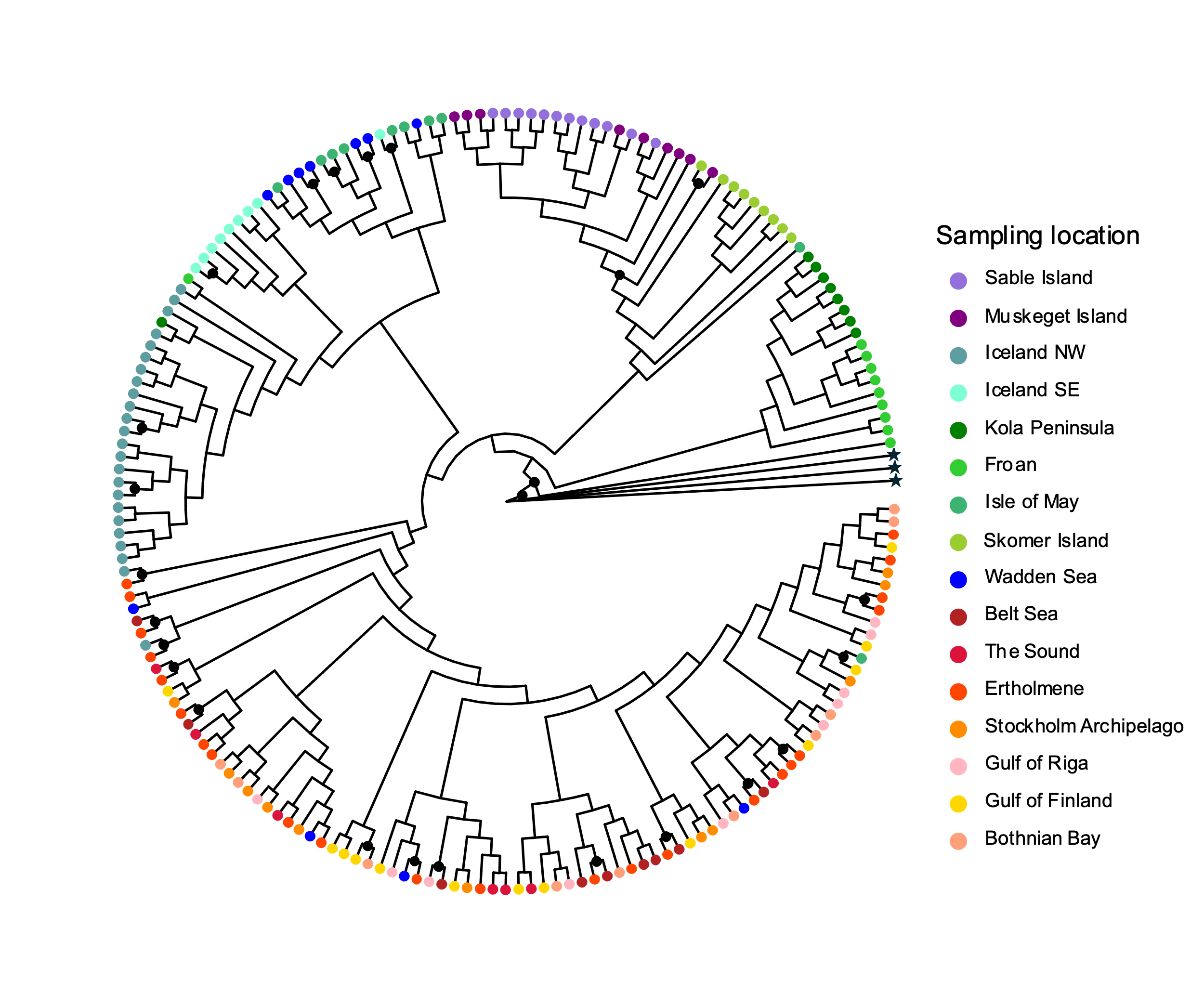
**Supplementary Figure S13:** Neighbour-joining tree based on a distance matrix calculated with ngsdist. Nodes supported with >90% bootstraps are indicated with a black circle. Three outgroup harbour seals are indicated with black star tip symbols.

**
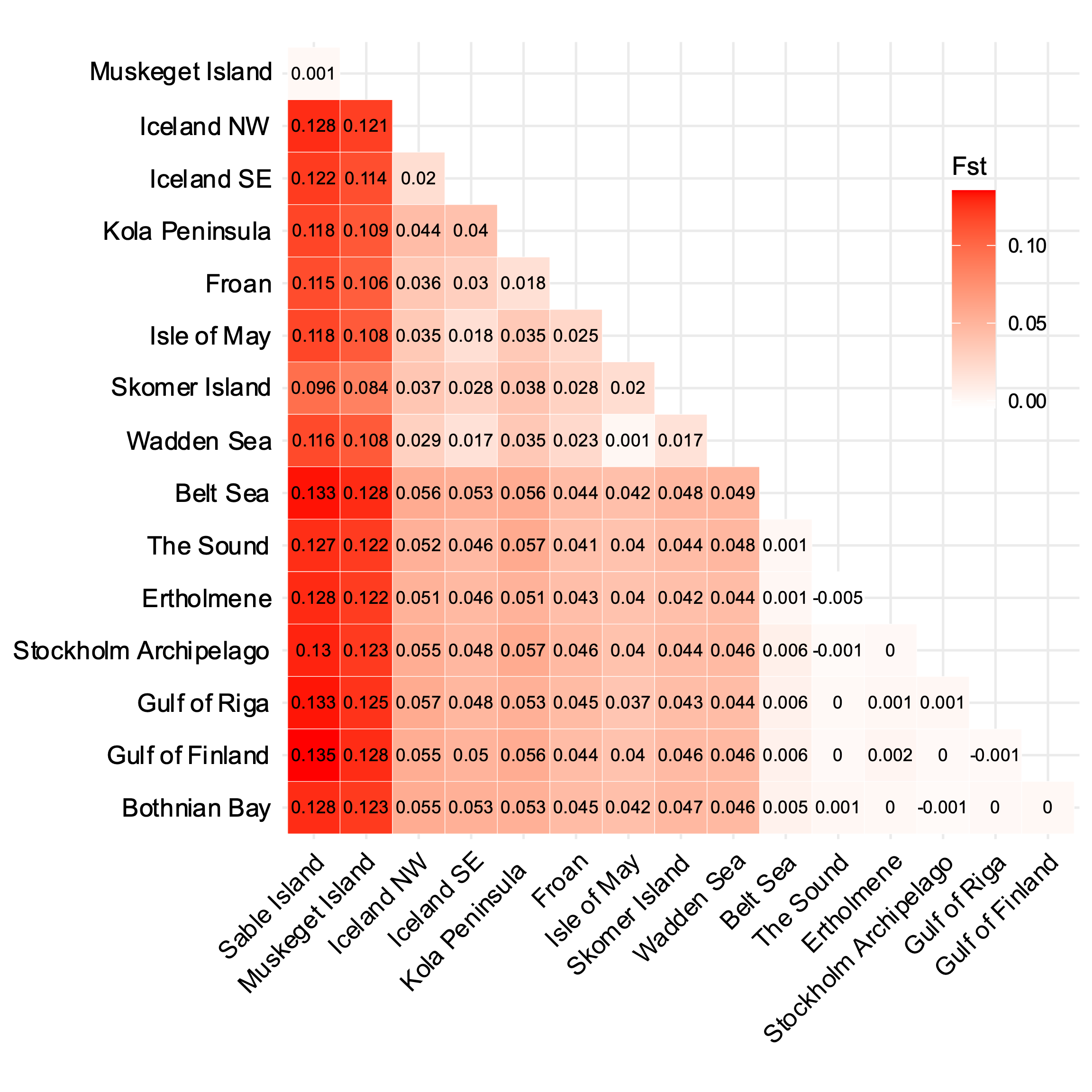
Supplementary Figure S14:** Genetic differentiation measured as *F*_ST_ between all localities. Kattegat was excluded due to a low sample size (n=2).


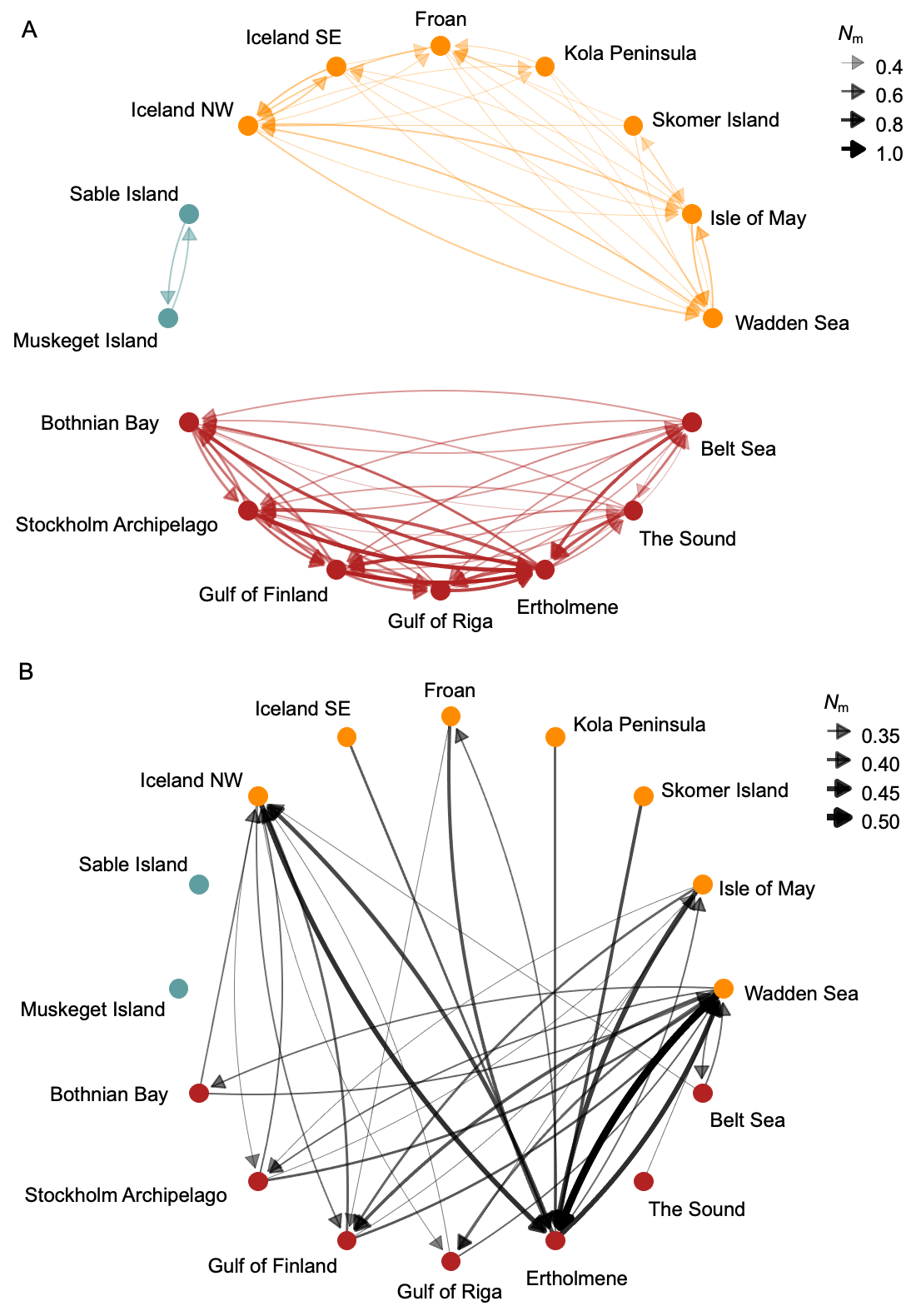


**Supplementary Figure S15:** Effective number of migrants (*N*_m_) from divMigrate decomposed into A) within and B) between region migrations. A minimum *N*_m_ filter of greater than 0.3 was used to assess the most meaningful patterns of migration.

**
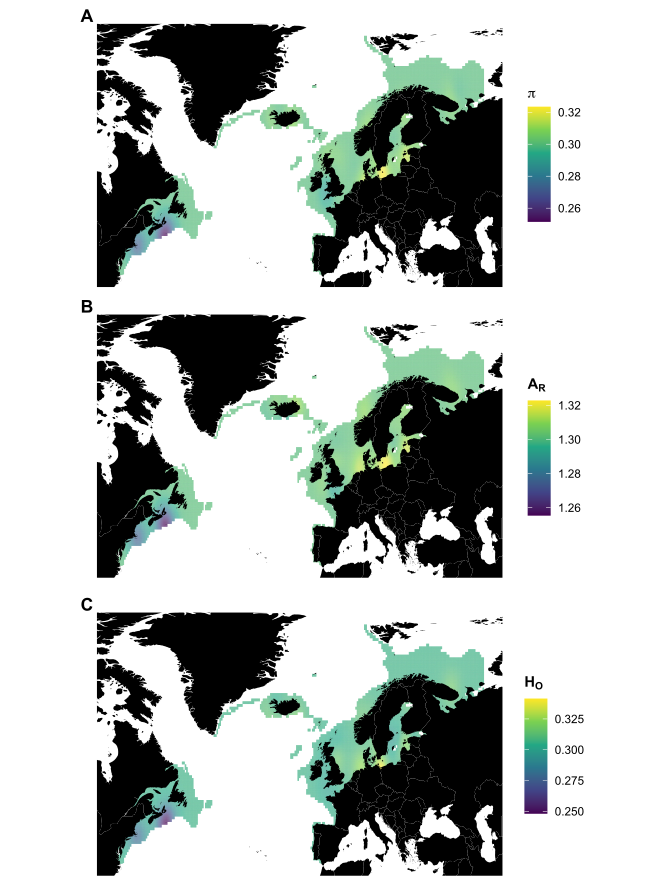
**

**Supplementary Figure S16:** Maps (A-C) of genetic diversity generated with the wingen R package for 185 gray seals and including 3793 SNPs. Genetic diversity metrics have been interpolated across gray seal suitable habitat. An overview of sampled colonies is presented in Figure 1A. Abbreviations: nucleotide diversity (𝛑), allelic richness (A_R_), heterozygosity (H_O_).

**
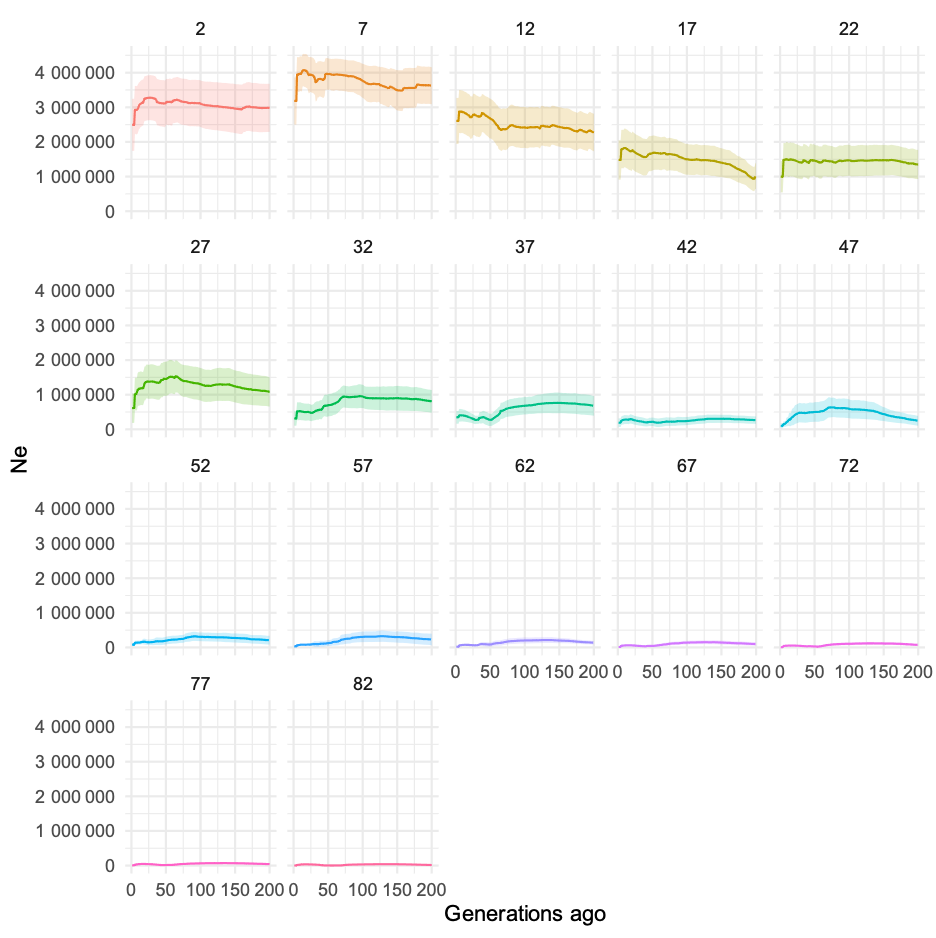
**

**Supplementary Figure S17:** Evaluating the effect of sample size on inference of *N*_e_ through GONE by downsampling the Baltic locality in increments of 5 animals from 82 to 2 animals. The subplot titles represent the number of seals chosen for each iteration. *N*_e_ becomes inflated beyond reasonable biological limits as the number of seals included decreases.

**Supplementary Table S1:**  Metadata and sequencing statistics for samples sequenced for this study.

| **Sample ID** | **Country location** | **Geographic location** | **Additional geographical information** | **Number of reads** | **Number of mapped reads** | **Depth of coverage (x)** | **Depth standard deviation** | **Included in final dataset** | **Age** | **Sex** | **Year** | **Month** | **Day** |
| --- | --- | --- | --- | --- | --- | --- | --- | --- | --- | --- | --- | --- | --- |
| HG007 | Denmark | Belt Sea | Rødsand | 2915869 | 0 | 0 | 0 | N | Unknown | Unknown | NA | NA | NA |
| HG008 | Denmark | Belt Sea | Rødsand | 2869726 | 1994147 | 21.0889 | 155.88 | N | Unknown | Unknown | NA | NA | NA |
| HG009 | Denmark | Belt Sea | Rødsand | 3645152 | 2930373 | 28.6583 | 188.1 | Y | Unknown | Unknown | NA | NA | NA |
| HG513 | Denmark | Belt Sea | Rødsand | 2216872 | 1006331 | 13.1463 | 77.21 | Y | Pup | Male | 2014 | 3 | 5 |
| HG514 | Denmark | Belt Sea | Rødsand | 2489538 | 1486773 | 16.575 | 105.97 | Y | Pup | Female | 2014 | 3 | 5 |
| HG515 | Denmark | Belt Sea | Rødsand | 2734447 | 1434954 | 15.472 | 99.18 | Y | Pup | Male | 2014 | 3 | 5 |
| HG516 | Denmark | Belt Sea | Rødsand | 1801643 | 1565348 | 18.4209 | 93.77 | Y | Pup | Male | 2014 | 3 | 5 |
| HG519 | Denmark | Ertholmene |  | 4946537 | 3795065 | 30.794 | 179.52 | Y | Unknown | Unknown | 2013 | 9 | 5 |
| HG521 | Denmark | Ertholmene |  | 790521 | 605650 | 8.3556 | 52.48 | Y | Juvenile | Female | 2014 | 7 | 17 |
| HG522 | Denmark | Ertholmene |  | 413523 | 331087 | 5.38253 | 30.02 | Y | Juvenile | Male | 2014 | 7 | 18 |
| HG523 | Denmark | Ertholmene |  | 9092297 | 1468572 | 16.5449 | 120.25 | Y | Juvenile | Female | 2014 | 7 | 19 |
| HG524 | Denmark | Ertholmene |  | 6547991 | 4906849 | 35.75 | 164.59 | Y | Unknown | Unknown | 2016 | 5 | 19 |
| HG525 | Denmark | Ertholmene |  | 3602678 | 2342623 | 23.0435 | 171.6 | Y | Unknown | Male | 2016 | 5 | 19 |
| HG526 | Denmark | Ertholmene |  | 4227208 | 3274949 | 28.5002 | 168.7 | Y | Unknown | Unknown | 2016 | 5 | 19 |
| HG527 | Denmark | Ertholmene |  | 3570854 | 3056974 | 28.8398 | 182.08 | Y | Unknown | Unknown | 2016 | 5 | 19 |
| HG528 | Germany | Wadden Sea | Helgoland | 1862220 | 1502375 | 14.6531 | 142.99 | Y | Unknown | Male | 2015 | 5 | 20 |
| HG529 | Germany | Wadden Sea | Helgoland | 413760 | 290114 | 5.08399 | 42.85 | Y | Unknown | Male | 2015 | 5 | 20 |
| HG530 | Germany | Wadden Sea | Helgoland | 1196758 | 976734 | 12.0028 | 92.12 | Y | Unknown | Male | 2015 | 5 | 20 |
| HG159 | Denmark | Ertholmene |  | 994141 | 821292 | 10.0744 | 96.74 | Y | Unknown | Unknown | 2012 | 10 | 18 |
| HG160 | Denmark | Ertholmene |  | 319276 | 268680 | 4.74677 | 29.06 | Y | Unknown | Unknown | 2012 | 10 | 18 |
| HG161 | Denmark | Ertholmene |  | 1923714 | 1739912 | 21.8426 | 159.27 | Y | Unknown | Unknown | 2012 | 10 | 18 |
| HG162 | Denmark | Ertholmene |  | 3067756 | 2410317 | 20.4149 | 171.91 | Y | Unknown | Unknown | 2012 | 10 | 18 |
| HG163 | Denmark | Ertholmene |  | 2526509 | 2201707 | 21.4436 | 179.4 | Y | Unknown | Unknown | 2012 | 10 | 18 |
| HG502 | Denmark | Ertholmene |  | 1388599 | 526039 | 7.85368 | 79.24 | Y | Unknown | Male | 2016 | 5 | 9 |
| HG503 | Denmark | Ertholmene |  | 1205056 | 857385 | 10.9801 | 107.34 | Y | Unknown | Female | 2016 | 5 | 9 |
| HG506 | Denmark | Ertholmene |  | 868103 | 698000 | 9.6695 | 91.52 | Y | Unknown | Female | 2016 | 5 | 10 |
| HG507 | Denmark | Ertholmene |  | 860571 | 483917 | 7.35293 | 74.71 | Y | Unknown | Male | 2016 | 5 | 10 |
| HG508 | Denmark | Ertholmene |  | 1211881 | 624887 | 8.58165 | 74.66 | Y | Unknown | Male | 2016 | 5 | 10 |
| HG509 | Denmark | Ertholmene |  | 1086274 | 610647 | 8.332 | 78.68 | Y | Unknown | Male | 2016 | 5 | 10 |
| HG510 | Denmark | Ertholmene |  | 798325 | 702016 | 9.06299 | 84.62 | Y | Unknown | Female | 2016 | 5 | 10 |
| HG511 | Denmark | Ertholmene |  | 1014307 | 691775 | 9.08724 | 87.02 | Y | Unknown | Male | 2016 | 5 | 10 |
| HG167 | Sweden | The Sound | Falsterbo | 365901 | 283694 | 4.83469 | 28.3 | Y | Juvenile | Male | 2012 | 11 | 14 |
| HG168 | Sweden | The Sound | Falsterbo | 466981 | 361593 | 5.37611 | 30.54 | Y | Juvenile | Male | 2012 | 11 | 13 |
| HG169 | Sweden | The Sound | Falsterbo | 406197 | 348806 | 5.67576 | 48.23 | Y | Juvenile | Female | 2012 | 12 | 6 |
| HG170 | Sweden | The Sound | Falsterbo | 4781432 | 2877276 | 23.1026 | 173.8 | Y | Juvenile | Male | 2012 | 12 | 6 |
| HG171 | Sweden | The Sound | Falsterbo | 12540125 | 10148558 | 73.8984 | 206.29 | Y | Juvenile | Male | 2012 | 12 | 7 |
| HG003 | Denmark | Kattegat | Anholt | 1136520 | 727479 | 9.48145 | 122.47 | N | Unknown | Unknown | 2008 | 4 | 29 |
| HG012 | Denmark | Kattegat | Sønderby Havn | 1746497 | 894267 | 10.9784 | 92.91 | Y | Unknown | Unknown | 2012 | 6 | 26 |
| HG500 | Denmark | Ertholmene | Christiansø | 438627 | 371494 | 5.89705 | 46.85 | Y | Unknown | Male | 2016 | 5 | 9 |
| HG121 | Denmark | Wadden Sea | Fanø | 383578 | 301588 | 4.98296 | 48.84 | Y | Unknown | Male | 2007 | 1 | 22 |
| HG501 | Denmark | Ertholmene |  | 561021 | 441485 | 7.05098 | 76.96 | Y | Unknown | Male | 2016 | 5 | 9 |
| HG126 | Denmark | Wadden Sea | Blåvand | 600896 | 495958 | 7.291 | 69.95 | Y | Unknown | Male | 2007 | 3 | 3 |
| HG127 | Denmark | Wadden Sea | Blåvand | 479773 | 427206 | 6.60861 | 70.41 | Y | Unknown | Male | 2007 | 3 | 7 |
| HG129 | Denmark | Wadden Sea | Blåvand | 356177 | 291383 | 5.09129 | 55.23 | Y | Unknown | Male | 2007 | 4 | 4 |
| HG545 | Iceland | Iceland NW | Breiðafjörður | 707928 | 653138 | 9.74662 | 91.9 | Y | Unknown | Female | 2008 | 6 | 9 |
| HG546 | Iceland | Iceland NW | Breiðafjörður | 574530 | 542927 | 8.68039 | 79.88 | Y | Unknown | Male | 2009 | 12 | 10 |
| HG577 | Iceland | Iceland NW | Eyjafjörður | 593347 | 475644 | 7.03805 | 65.02 | N | Unknown | Male | 2009 | 4 | 20 |
| HG138 | Denmark | Wadden Sea | Skallingen | 344959 | 301519 | 5.05717 | 46.47 | Y | Unknown | Male | 2008 | 12 | 23 |
| HG578 | Iceland | Iceland NW | Breiðafjörður | 438946 | 391830 | 6.20229 | 53.84 | Y | Unknown | Male | 2008 | 8 | 15 |
| HG140 | Denmark | Wadden Sea | Blåvand | 663370 | 599174 | 8.47129 | 86.67 | Y | Unknown | Female | 2009 | 2 | 1 |
| HG543 | Iceland | Iceland NW | Breiðafjörður | 486403 | 362265 | 5.9233 | 62.21 | Y | Unknown | Male | 2009 | 5 | 30 |
| HG544 | Iceland | Iceland NW | Breiðafjörður | 524596 | 470717 | 7.31279 | 70.41 | Y | Unknown | Female | 2008 | 5 | 4 |
| HG547 | Iceland | Iceland NW | Strandir | 2659452 | 641688 | 8.83483 | 91.81 | Y | Unknown | Female | 2009 | 5 | 1 |
| HG548 | Iceland | Iceland SE | Djúpivogur | 2580525 | 1888287 | 18.5139 | 171.58 | N | Unknown | Female | 2009 | 10 | 26 |
| HG549 | Iceland | Iceland SE | Djúpivogur | 1142996 | 937994 | 13.0787 | 106.06 | Y | Unknown | Female | 2009 | 10 | 26 |
| HG550 | Iceland | Iceland SE | Djúpavogur | 1891196 | 777465 | 10.5113 | 92.42 | Y | Unknown | Female | 2009 | 10 | 26 |
| HG551 | Iceland | Iceland NW | Strandir | 1766347 | 1071971 | 13.8307 | 128.43 | Y | Unknown | Male | 2009 | 6 | 1 |
| HG552 | Iceland | Iceland SE | Djúpivogur | 2086373 | 664469 | 9.38615 | 87.11 | Y | Unknown | Female | 2009 | 10 | 26 |
| HG553 | Iceland | Iceland NW | Strandir | 2739506 | 2393851 | 23.6385 | 165.69 | Y | Unknown | Male | 2009 | 5 | 1 |
| HG554 | Iceland | Iceland NW | Strandir | 2329929 | 939991 | 11.0711 | 104.16 | Y | Unknown | Male | 2009 | 5 | 1 |
| HG555 | Iceland | Iceland NW | Skagafjörður | 2774819 | 2380745 | 23.5495 | 166.65 | Y | Unknown | Female | 2009 | 5 | 15 |
| HG556 | Iceland | Iceland NW | Skagafjörður | 1686162 | 1394788 | 15.3876 | 130.79 | Y | Unknown | Female | 2009 | 5 | 18 |
| HG557 | Iceland | Iceland NW | Skagafjörður | 4137805 | 3534645 | 28.0792 | 170.17 | N | Unknown | Female | 2009 | 5 | 15 |
| HG558 | Iceland | Iceland NW | Húnaflói | 6738199 | 2753039 | 24.5614 | 166.79 | Y | Unknown | Female | 2009 | 6 | 9 |
| HG559 | Iceland | Iceland NW | Húnaflói | 2428106 | 1880906 | 17.371 | 124.95 | N | Unknown | Unknown | 2010 | 5 | 20 |
| HG560 | Iceland | Iceland NW | Húnaflói | 3293273 | 2290008 | 21.6692 | 164.86 | Y | Unknown | Unknown | 2010 | 5 | 5 |
| HG561 | Iceland | Iceland NW | Húnaflói | 2623303 | 1953016 | 20.7243 | 156.7 | Y | Unknown | Male | 2009 | 6 | 3 |
| HG562 | Iceland | Iceland NW | Húnaflói | 1314027 | 1087757 | 12.9458 | 118.17 | Y | Unknown | Female | 2009 | 11 | 1 |
| HG563 | Iceland | Iceland NW | Húnaflói | 1330521 | 1106755 | 14.4358 | 131.43 | Y | Unknown | Unknown | 10 | 5 | 25 |
| HG564 | Iceland | Iceland NW | Húnaflói | 1549048 | 1283015 | 14.4974 | 134.57 | Y | Unknown | Male | 2009 | 4 | 20 |
| HG565 | Iceland | Iceland NW | Húnaflói | 1396314 | 1184517 | 13.1708 | 125.44 | Y | Unknown | Female | 2008 | 4 | 21 |
| HG566 | Iceland | Iceland NW | Kaldbaksvik | 619808 | 535827 | 7.74581 | 80.96 | Y | Unknown | Male | 2009 | 3 | 26 |
| HG567 | Iceland | Iceland SE | Djúpavogur | 465828 | 383411 | 6.26045 | 60.07 | Y | Unknown | Male | 2009 | 10 | 26 |
| HG568 | Iceland | Iceland SE | Djúpivogur | 412996 | 359601 | 6.064 | 53.59 | Y | Unknown | Male | 2009 | 10 | 26 |
| HG569 | Iceland | Iceland SE | Djúpivogur | 684351 | 590269 | 8.00271 | 84.11 | Y | Unknown | Female | 2009 | 10 | 26 |
| HG570 | Iceland | Iceland SE | Djúpivogur | 1151099 | 1022113 | 13.3222 | 130.55 | Y | Unknown | Female | 2009 | 10 | 26 |
| HG571 | Iceland | Iceland SE | Djúpivogur | 995125 | 779198 | 10.2049 | 90.22 | Y | Unknown | Female | 2009 | 10 | 26 |
| HG572 | Iceland | Iceland NW | Patreksfjörður | 370827 | 309749 | 5.14196 | 43.63 | Y | Unknown | Male | 2009 | 5 | 30 |
| HG573 | Iceland | Iceland SE | Djúpivogur | 883515 | 763865 | 9.69782 | 89.23 | Y | Unknown | Male | 2009 | 10 | 26 |
| HG574 | Iceland | Iceland NW | Patreksfjörður | 680797 | 582232 | 8.07782 | 77.74 | Y | Unknown | Male | 2009 | 5 | 30 |
| HG575 | Iceland | Iceland NW | Breiðafjörður | 352530 | 278957 | 4.79046 | 42.75 | Y | Unknown | Female | 2008 | 5 | 30 |
| HG579 | Iceland | Iceland NW | Breiðafjörður | 2994837 | 976185 | 14.2638 | 120.62 | Y | Unknown | Male | 2009 | 9 | 6 |
| HG580 | Iceland | Iceland NW | Breiðafjörður | 2123938 | 716987 | 11.1291 | 84.1 | N | Unknown | Female | 2008 | 8 | 14 |
| HG601 | Russia | Kola Peninsula |  | 779113 | 629176 | 10.537 | 83.41 | Y | Unknown | Unknown | 1994 | 11 | 20 |
| HG602 | Russia | Kola Peninsula |  | 1546229 | 546593 | 9.71876 | 81.07 | Y | Unknown | Unknown | 1994 | 11 | 24 |
| HG603 | Russia | Kola Peninsula |  | 1597918 | 771701 | 11.9612 | 117.56 | Y | Unknown | Unknown | 1994 | 11 | 21 |
| HG604 | Russia | Kola Peninsula |  | 1570978 | 776776 | 12.3823 | 97.19 | Y | Unknown | Unknown | 1994 | 11 | 23 |
| HG605 | Russia | Kola Peninsula |  | 1199030 | 1056202 | 15.6935 | 126.09 | Y | Unknown | Unknown | 1994 | 11 | 23 |
| HG606 | Russia | Kola Peninsula |  | 2326891 | 1463153 | 20.507 | 168.87 | Y | Unknown | Unknown | 1994 | 11 | 23 |
| HG607 | Russia | Kola Peninsula |  | 1345785 | 1137368 | 16.8 | 129.63 | Y | Unknown | Unknown | 1994 | 11 | 24 |
| HG608 | Russia | Kola Peninsula |  | 1276650 | 1057574 | 15.897 | 125.74 | Y | Unknown | Unknown | 1994 | 11 | 22 |
| HG609 | Russia | Kola Peninsula |  | 2024798 | 1712538 | 21.8062 | 173.88 | N | Unknown | Unknown | 1994 | 11 | 22 |
| HG610 | Russia | Kola Peninsula |  | 3701335 | 618771 | 10.0756 | 88.1 | Y | Unknown | Unknown | 1994 | 11 | 23 |
| HG611 | Norway | Froan |  | 2653516 | 2016840 | 23.8087 | 176.33 | Y | Unknown | Unknown | 1996 | NA | NA |
| HG612 | Norway | Froan |  | 3357895 | 2442863 | 28.721 | 182.61 | Y | Unknown | Unknown | 1996 | NA | NA |
| HG613 | Norway | Froan |  | 4411729 | 3547468 | 37.764 | 187.64 | Y | Unknown | Unknown | 1996 | NA | NA |
| HG614 | Norway | Froan |  | 2726407 | 2355958 | 29.2417 | 194.74 | Y | Unknown | Unknown | 1996 | NA | NA |
| HG615 | Norway | Froan |  | 1633536 | 1394638 | 19.399 | 160.47 | Y | Unknown | Unknown | 1996 | NA | NA |
| HG616 | Norway | Froan |  | 1310495 | 1129568 | 16.4653 | 143.85 | Y | Unknown | Unknown | 1996 | NA | NA |
| HG617 | Norway | Froan |  | 767290 | 656614 | 11.217 | 93.14 | Y | Unknown | Unknown | 1996 | NA | NA |
| HG618 | Norway | Froan |  | 797522 | 700995 | 11.5201 | 85.8 | Y | Unknown | Unknown | 1996 | NA | NA |
| HG619 | Norway | Froan |  | 1184629 | 1027508 | 15.4005 | 122.38 | Y | Unknown | Unknown | 1996 | NA | NA |
| HG620 | Norway | Froan |  | 690392 | 624757 | 10.9085 | 94.93 | Y | Unknown | Unknown | 1996 | NA | NA |
| HG148 | UK | Isle of May |  | 368399 | 324816 | 6.10984 | 53.15 | Y | Yearling | Unknown | 2011 | 11 | 28 |
| HG005 | Denmark | The Sound | Køge Bugt | 784495 | 712278 | 11.2857 | 89.58 | Y | Unknown | Unknown | NA | NA | NA |
| HG142 | UK | Isle of May |  | 7560013 | 6287374 | 60.1823 | 205.89 | Y | Yearling | Unknown | 2011 | 11 | 15 |
| HG143 | UK | Isle of May |  | 354096 | 285656 | 5.25354 | 43.65 | Y | Yearling | Unknown | 2011 | 11 | 15 |
| HG144 | UK | Isle of May |  | 1318792 | 1187416 | 16.366 | 140.98 | Y | Yearling | Unknown | 2011 | 11 | 15 |
| HG145 | UK | Isle of May |  | 849583 | 730539 | 10.997 | 100.52 | Y | Yearling | Unknown | 2011 | 11 | 15 |
| HG006 | Denmark | The Sound | Klintholm | 2920887 | 791181 | 11.7976 | 98.38 | Y | Unknown | Unknown | NA | NA | NA |
| HG151 | UK | Isle of May |  | 963149 | 194602 | 3.90338 | 19.59 | Y | Yearling | Unknown | 2011 | 12 | 1 |
| HG152 | UK | Isle of May |  | 3546062 | 2668890 | 29.3508 | 196.93 | Y | Yearling | Unknown | 2011 | 12 | 2 |
| HG153 | UK | Isle of May |  | 1813644 | 734160 | 11.0883 | 92.25 | Y | Yearling | Unknown | 2011 | 12 | 2 |
| HG156 | UK | Isle of May | Farne island | 1178338 | 568033 | 8.83858 | 51.04 | Y | Pup | Unknown | 2012 | NA | NA |
| HG157 | UK | Isle of May | Farne island | 2470806 | 1433354 | 18.8358 | 138.69 | Y | Pup | Unknown | 2012 | NA | NA |
| HG673 | Finland | Bothnian Bay | Bothnian Bay | 1121558 | 955302 | 13.5918 | 108.9 | Y | Unknown | Female | 2017 | NA | NA |
| HG576 | Iceland | Iceland NW | Breiðafjörður | 2445664 | 1365516 | 18.3405 | 131.14 | Y | Unknown | Male | 2008 | 5 | 7 |
| HG300 | Denmark | Belt Sea | Rødsand | 2761726 | 2369328 | 26.3193 | 193.72 | Y | Pup | Male | 2013 | 3 | 4 |
| HG301 | Denmark | Belt Sea | Rødsand | 1249982 | 1003072 | 14.5242 | 95.84 | Y | Pup | Female | 2013 | 3 | 4 |
| HG517 | Denmark | Wadden Sea | Limfjord, Rønland Sandø | 6264523 | 3003991 | 34.5568 | 182.05 | Y | Pup | Unknown | 2015 | 7 | NA |
| HG088 | Denmark | Kattegat | Grenå | 3259153 | 2709622 | 29.1221 | 178.96 | Y | Unknown | Female | 1999 | 1 | 19 |
| HG102 | Denmark | Belt Sea | Nordals | 837480 | 198980 | 3.85051 | 20.92 | Y | Unknown | Female | 2000 | NA | NA |
| HG123 | Denmark | Wadden Sea | Nymindegab | 3631511 | 2787335 | 31.5637 | 192.41 | Y | Unknown | Male | 2007 | 12 | 24 |
| HG635 | Sweden | Stockholm Archipelago |  | 4137361 | 3581304 | 36.8195 | 188.1 | Y | Unknown | Unknown | 2016 | NA | NA |
| HG668 | Finland | Bothnian Bay |  | 1231885 | 1013194 | 14.4184 | 131.82 | Y | Unknown | Female | 2017 | NA | NA |
| HG669 | Finland | Bothnian Bay |  | 1138973 | 955044 | 13.5397 | 115.22 | Y | Unknown | Female | 2017 | NA | NA |
| HG670 | Finland | Bothnian Bay |  | 524329 | 447170 | 7.41447 | 67.56 | Y | Unknown | Female | 2017 | NA | NA |
| HG671 | Finland | Bothnian Bay |  | 834944 | 724163 | 10.8927 | 89.58 | Y | Unknown | Female | 2017 | NA | NA |
| HG625 | Sweden | Stockholm Archipelago |  | 3284016 | 2809695 | 30.7317 | 189.88 | Y | Unknown | Unknown | 2010 | NA | NA |
| HG626 | Sweden | Stockholm Archipelago |  | 421476 | 373070 | 6.28075 | 49.91 | Y | Unknown | Unknown | 2010 | NA | NA |
| HG627 | Sweden | Stockholm Archipelago |  | 1024780 | 912862 | 13.0029 | 108.6 | Y | Unknown | Unknown | 2010 | NA | NA |
| HG628 | Sweden | Stockholm Archipelago |  | 1076873 | 972101 | 13.9747 | 109.91 | Y | Unknown | Unknown | 2011 | NA | NA |
| HG629 | Sweden | Stockholm Archipelago |  | 1623710 | 1347623 | 18.4043 | 137.7 | Y | Unknown | Unknown | 2011 | NA | NA |
| HG630 | Sweden | Stockholm Archipelago |  | 428772 | 372960 | 6.52324 | 56.57 | Y | Unknown | Unknown | 2012 | NA | NA |
| HG631 | Sweden | Stockholm Archipelago |  | 308150 | 249347 | 4.71667 | 30.39 | Y | Unknown | Unknown | 2012 | NA | NA |
| HG632 | Sweden | Stockholm Archipelago |  | 467436 | 391879 | 6.71922 | 51.26 | Y | Unknown | Unknown | 2013 | NA | NA |
| HG633 | Sweden | Stockholm Archipelago |  | 858750 | 703382 | 10.7857 | 99.51 | Y | Unknown | Unknown | 2015 | NA | NA |
| HG634 | Sweden | Stockholm Archipelago |  | 623920 | 481335 | 7.88031 | 77.87 | Y | Unknown | Unknown | 2016 | NA | NA |
| HG674 | Finland | Gulf of Finland |  | 4356663 | 1460246 | 17.9979 | 150.34 | Y | Unknown | Female | 2017 | NA | NA |
| HG675 | Finland | Gulf of Finland |  | 4640454 | 2573797 | 26.4475 | 185.35 | Y | Unknown | Male | 2017 | NA | NA |
| HG676 | Finland | Gulf of Finland |  | 1591919 | 1237000 | 16.4788 | 118.63 | Y | Unknown | Female | 2017 | NA | NA |
| HG666 | Finland | Gulf of Finland |  | 2585660 | 861771 | 12.4167 | 93.33 | Y | Unknown | Female | 2017 | NA | NA |
| HG641 | Estonia | Gulf of Riga |  | 4435960 | 3119137 | 32.2121 | 190.16 | Y | Pup | Male | 2016 | 3 | 6 |
| HG642 | Estonia | Gulf of Riga |  | 4760172 | 2387718 | 27.5703 | 180.51 | Y | Pup | Female | 2016 | 3 | 6 |
| HG643 | Estonia | Gulf of Riga |  | 5100495 | 4493539 | 43.1581 | 182.78 | Y | Pup | Female | 2016 | 3 | 6 |
| HG644 | Estonia | Gulf of Riga |  | 3988715 | 2736005 | 31.9264 | 180.57 | Y | Pup | Female | 2016 | 3 | 6 |
| HG645 | Estonia | Gulf of Riga |  | 2052170 | 1763394 | 21.8029 | 153.88 | Y | Pup | Female | 2016 | 3 | 6 |
| HG646 | Estonia | Gulf of Riga |  | 897132 | 708314 | 10.6424 | 79.45 | Y | Pup | Male | 2016 | 3 | 6 |
| HG647 | Estonia | Gulf of Riga |  | 1920399 | 1559906 | 20.4744 | 133.16 | Y | Pup | Female | 2016 | 3 | 6 |
| HG648 | Estonia | Gulf of Riga |  | 5363573 | 1498796 | 19.2222 | 120.66 | Y | Pup | Male | 2016 | 3 | 6 |
| HG649 | Estonia | Gulf of Riga |  | 2577801 | 2100759 | 24.3618 | 160.19 | Y | Pup | Male | 2016 | 3 | 6 |
| HG650 | Estonia | Gulf of Riga |  | 3279359 | 2293909 | 25.8886 | 174.06 | Y | Pup | Male | 2016 | 3 | 6 |
| HG651 | Finland | Bothnian Bay |  | 1403209 | 926998 | 13.0118 | 101.19 | Y | Unknown | Male | 2017 | NA | NA |
| HG652 | Finland | Gulf of Finland |  | 2410071 | 2092828 | 23.7901 | 167.5 | Y | Unknown | Male | 2017 | NA | NA |
| HG653 | Finland | Gulf of Finland |  | 1416541 | 1170694 | 15.0723 | 145.65 | Y | Unknown | Female | 2017 | NA | NA |
| HG654 | Finland | Gulf of Finland |  | 865807 | 735188 | 10.9318 | 95.28 | Y | Unknown | Female | 2017 | NA | NA |
| HG655 | Finland | Gulf of Finland |  | 821994 | 705117 | 10.579 | 82.16 | Y | Unknown | Female | 2017 | NA | NA |
| HG656 | Finland | Bothnian Bay |  | 1026210 | 898374 | 12.6317 | 106.74 | Y | Unknown | Female | 2017 | NA | NA |
| HG657 | Finland | Bothnian Bay |  | 1572468 | 1325956 | 17.2165 | 135.29 | Y | Unknown | Male | 2017 | NA | NA |
| HG658 | Finland | Bothnian Bay |  | 1011483 | 904652 | 12.7573 | 92.58 | Y | Unknown | Female | 2017 | NA | NA |
| HG659 | Finland | Gulf of Finland |  | 1152823 | 1028044 | 14.2065 | 96.16 | Y | Unknown | Female | 2017 | NA | NA |
| HG660 | Finland | Gulf of Finland |  | 2293703 | 2078479 | 25.2353 | 168.26 | Y | Unknown | Female | 2017 | NA | NA |
| HG662 | Finland | Gulf of Finland |  | 1771554 | 1578350 | 19.8908 | 140.11 | Y | Unknown | Female | 2017 | NA | NA |
| HG663 | Finland | Gulf of Finland |  | 815228 | 704104 | 10.3956 | 80.65 | Y | Unknown | Male | 2017 | NA | NA |
| HG664 | Finland | Gulf of Finland |  | 1128283 | 928321 | 12.9966 | 102.33 | Y | Unknown | Female | 2017 | NA | NA |
| HG672 | Finland | Bothnian Bay |  | 656949 | 562977 | 8.65457 | 82.5 | Y | Unknown | Female | 2017 | NA | NA |
| HG588 | UK | Skomer Island |  | 2098906 | 65060 | 1.98101 | 8.4 | N | Unknown | Unknown | 2015 | 11 | 15 |
| HG589 | UK | Skomer Island |  | 1544352 | 135884 | 3.04832 | 18.63 | Y | Unknown | Unknown | 2015 | 11 | 15 |
| HG590 | UK | Skomer Island |  | 270757 | 190667 | 4.08865 | 30.76 | Y | Unknown | Unknown | 2015 | 11 | 17 |
| HG591 | UK | Skomer Island |  | 1514487 | 320945 | 5.5341 | 48.11 | Y | Unknown | Unknown | 2015 | 9 | 30 |
| HG592 | UK | Skomer Island |  | 1337029 | 391094 | 7.08282 | 59.06 | Y | Unknown | Unknown | 2015 | 9 | 30 |
| HG593 | UK | Skomer Island |  | 1878566 | 443599 | 7.6918 | 66.73 | Y | Unknown | Unknown | 2015 | 10 | 18 |
| HG594 | UK | Skomer Island |  | 1187832 | 1003726 | 13.7674 | 117.25 | N | Unknown | Unknown | 2015 | 10 | 18 |
| HG595 | UK | Skomer Island |  | 1328028 | 305368 | 4.95136 | 37.63 | Y | Unknown | Unknown | 2015 | 10 | 22 |
| HG596 | UK | Skomer Island |  | 971202 | 804495 | 12.1724 | 101.43 | Y | Unknown | Unknown | 2015 | 10 | 8 |
| HG597 | UK | Skomer Island |  | 191331 | 38208 | 1.67182 | 6.05 | N | Unknown | Unknown | 2015 | 10 | 22 |
| HG598 | UK | Skomer Island |  | 450292 | 299099 | 4.87555 | 41.24 | Y | Unknown | Unknown | 2015 | 10 | 14 |
| HG599 | UK | Skomer Island |  | 3856219 | 773528 | 10.5541 | 90.34 | Y | Unknown | Unknown | 2015 | 10 | NA |
| HG154 | Denmark | Belt Sea | Rødsand | 631580 | 463642 | 7.7312 | 59.26 | Y | Unknown | Unknown | 2009 | 10 | 31 |
| HG691 | Faroe Islands | Faroe Islands |  | 818594 | 28526 | 1.61487 | 5.35 | N | Unknown | Unknown | NA | NA | NA |
| HG692 | Faroe Islands | Faroe Islands |  | 114677 | 32235 | 1.57914 | 4.98 | N | Unknown | Unknown | NA | NA | NA |
| HG677 | Faroe Islands | Faroe Islands |  | 34876 | 12227 | 1.3308 | 3 | N | Unknown | Unknown | NA | NA | NA |
| HG678 | Faroe Islands | Faroe Islands |  | 13011 | 9069 | 1.37171 | 2.7 | N | Unknown | Unknown | NA | NA | NA |
| HG679 | Faroe Islands | Faroe Islands |  | 12485 | 8219 | 1.28228 | 2.47 | N | Unknown | Unknown | NA | NA | NA |
| HG680 | Faroe Islands | Faroe Islands |  | 17098 | 13492 | 1.37998 | 3.52 | N | Unknown | Unknown | NA | NA | NA |
| HG681 | Faroe Islands | Faroe Islands |  | 14538 | 9928 | 1.31153 | 2.9 | N | Unknown | Unknown | NA | NA | NA |
| HG682 | Faroe Islands | Faroe Islands |  | 23509 | 17343 | 1.51725 | 4.22 | N | Unknown | Unknown | NA | NA | NA |
| HG683 | Faroe Islands | Faroe Islands |  | 19765 | 7718 | 1.36029 | 2.36 | N | Unknown | Unknown | NA | NA | NA |
| HG684 | Faroe Islands | Faroe Islands |  | 12636 | 9781 | 1.44624 | 3.95 | N | Unknown | Unknown | NA | NA | NA |
| HG685 | Faroe Islands | Faroe Islands |  | 38327 | 19162 | 1.62389 | 7.57 | N | Unknown | Unknown | NA | NA | NA |
| HG686 | Faroe Islands | Faroe Islands |  | 26918 | 18250 | 1.67725 | 7.39 | N | Unknown | Unknown | NA | NA | NA |
| HG687 | Faroe Islands | Faroe Islands |  | 64484 | 33178 | 1.7679 | 9.65 | N | Unknown | Unknown | NA | NA | NA |
| HG688 | Faroe Islands | Faroe Islands |  | 32319 | 24131 | 1.68619 | 9.06 | N | Unknown | Unknown | NA | NA | NA |
| HG689 | Faroe Islands | Faroe Islands |  | 34871 | 13844 | 1.60281 | 6.91 | N | Unknown | Unknown | NA | NA | NA |
| HG690 | Faroe Islands | Faroe Islands |  | 2052 | 882 | 1.25445 | 0.68 | N | Unknown | Unknown | NA | NA | NA |
| UMB015 | US | USA | Muskeget Island | 2570662 | 729238 | 11.2714 | 84.73 | Y | Pup | M | 2002 | 2 | 22 |
| UMB004 | US | USA | Muskeget Island | 3431416 | 1837755 | 24.2281 | 154.86 | Y | Pup | F | 2002 | 2 | 21 |
| Hg805 | US | USA | Muskeget Island | 932320 | 753930 | 11.665 | 76.23 | Y | Pup | F | 2016 | 1 | 15 |
| Hg849 | US | USA | Muskeget Island | 2378911 | 1147712 | 16.5805 | 130.7 | Y | Pup | M | 2016 | 1 | 15 |
| Hg812 | US | USA | Muskeget Island | 1172619 | 446992 | 7.75057 | 59.62 | Y | Pup | M | 2016 | 1 | 15 |
| Hg824 | US | USA | Muskeget Island | 2865401 | 1040493 | 15.3073 | 123.74 | Y | Pup | M | 2016 | 1 | 28 |
| Hg358 | US | USA | Muskeget Island | 1164367 | 1000462 | 14.5495 | 109.92 | Y | Pup | F | 2015 | 1 | 12 |
| Hg360 | US | USA | Muskeget Island | 1864625 | 1085181 | 15.3308 | 131.09 | Y | Pup | F | 2015 | 1 | 12 |
| Hg377 | US | USA | Muskeget Island | 1120466 | 941859 | 14.0521 | 112.02 | Y | Pup | F | 2015 | 1 | 13 |
| B141 | Canada | Sable Island |  | 1242365 | 1005746 | 14.7499 | 100.44 | Y | Adult | F | 2008 | 1 | 8 |
| B152 | Canada | Sable Island |  | 841268 | 673168 | 10.851 | 92.47 | Y | Adult | F | 2004 | 1 | NA |
| F423 | Canada | Sable Island |  | 5782631 | 2616151 | 32.5665 | 168.74 | Y | Adult | F | 2006 | 1 | 8 |
| F126 | Canada | Sable Island |  | 926455 | 732460 | 11.6123 | 84.84 | Y | Adult | F | 2002 | 1 | NA |
| 6X5 | Canada | Sable Island |  | 1389762 | 566091 | 9.14331 | 71.07 | Y | Pup | M | 1998 | 1 | 31 |
| 1X2 | Canada | Sable Island |  | 1019538 | 563555 | 8.97795 | 66.79 | Y | Pup | M | 1998 | 1 | 30 |
| S04-06 | Canada | Sable Island |  | 756590 | 602461 | 9.87365 | 64.61 | Y | Pup | F | 2004 | 1 | NA |
| S24 | Canada | Sable Island |  | 403556 | 327632 | 6.04718 | 39.42 | Y | Pup | F | 2015 | 1 | NA |
| S31 | Canada | Sable Island |  | 234470 | 195363 | 4.24376 | 28.33 | Y | Pup | F | 2015 | 1 | NA |
| S51 | Canada | Sable Island |  | 352717 | 296449 | 5.60769 | 39.23 | Y | Pup | F | 2015 | 1 | NA |
| S69 | Canada | Sable Island |  | 528335 | 465605 | 7.92314 | 61.07 | Y | Pup | M | 2015 | 1 | NA |
| S70 | Canada | Sable Island |  | 288611 | 245539 | 4.74668 | 31.54 | Y | Pup | F | 2015 | 1 | NA |
| D01464 (Harbour seal) | US | USA | NE US | 334323 | 298007 | 5.79553 | 58.56 | N | NA | M | 1995 | 10 | 17 |
| D01638 (Harbour seal) | US | USA | NE US | 688765 | 551434 | 9.71322 | 109.65 | N | NA | U | 1993 | 11 | NA |
| D05021 (Harbour seal) | US | USA | NE US | 450580 | 394954 | 7.74466 | 102.31 | N | NA | M | 2004 | 7 | 24 |
| D05320 (Harbour seal) | US | USA | NE US | 1290890 | 1143704 | 16.1644 | 157.47 | N | NA | M | 2004 | 9 | 13 |
| D00579 (Harbour seal) | US | USA | NE US | 1022482 | 880533 | 13.5581 | 144.02 | N | NA | U | 2015 | 12 | 13 |
| D05712 (Harbour seal) | US | USA | NE US | 536787 | 422308 | 7.8918 | 95.57 | N | NA | U | 2015 | 10 | 22 |

**Supplementary Table S2:** Filters and the number (#) of SNPs remaining after each quality control measure for final and exploratory datasets.

| **Dataset** | **Number of SNPs following MAF 0.05** | **Number of SNPs following HWE filter** | **Number of SNPS following global depth filter** |
| --- | --- | --- | --- |
| Global | 4873 | 4764 | 3812 |
| NE Atlantic-Baltic Sea | 4892 | 4781 | 3825 |
| NE Atlantic | 4727 | 4687 | 3750 |
| Baltic | 4911 | 4858 | 3886 |
